# Inhibition of minor intron splicing reduces Na^+^ and Ca^2+^ channel expression and function in cardiomyocytes

**DOI:** 10.1242/jcs.259191

**Published:** 2022-01-07

**Authors:** Pablo Montañés-Agudo, Simona Casini, Simona Aufiero, Auriane C. Ernault, Ingeborg van der Made, Yigal M. Pinto, Carol Ann Remme, Esther E. Creemers

**Affiliations:** 1 Departments of Experimental Cardiology, Amsterdam UMC, location AMC, 1105 AZ, Amsterdam, The Netherlands; 2 Clinical Epidemiology, Biostatistics and Bioinformatics, Amsterdam UMC, location AMC, 1105 AZ, Amsterdam, The Netherlands

**Keywords:** Heart, Intron retention, Ion channels, Minor spliceosome, Splicing

## Abstract

Eukaryotic genomes contain a tiny subset of ‘minor class’ introns with unique sequence elements that require their own splicing machinery. These minor introns are present in certain gene families with specific functions, such as voltage-gated Na^+^ and voltage-gated Ca^2+^ channels. Removal of minor introns by the minor spliceosome has been proposed as a post-transcriptional regulatory layer, which remains unexplored in the heart. Here, we investigate whether the minor spliceosome regulates electrophysiological properties of cardiomyocytes by knocking down the essential minor spliceosome small nuclear snRNA component *U6atac* in neonatal rat ventricular myocytes. Loss of *U6atac* led to robust minor intron retention within *Scn5a* and *Cacna1c*, resulting in reduced protein levels of Na_v_1.5 and Ca_v_1.2 channels. Functional consequences were studied through patch-clamp analysis, and revealed reduced Na^+^ and L-type Ca^2+^ currents after loss of *U6atac*. In conclusion, minor intron splicing modulates voltage-dependent ion channel expression and function in cardiomyocytes. This may be of particular relevance in situations in which minor splicing activity changes, such as in genetic diseases affecting minor spliceosome components, or in acquired diseases in which minor spliceosome components are dysregulated, such as heart failure.

## Abstract (precis)

**Summary:** Knockdown of minor spliceosome component *U6atac* in cardiomyocytes reveals that expression of the Na^+^ channel *Scn5a* and the L-type Ca^2+^ channel *Cacna1c* critically depend on minor intron splicing.

## INTRODUCTION

The accurate removal of introns by pre-mRNA splicing is a fundamental step in eukaryotic gene expression. Two types of introns coexist in eukaryotes – major introns and minor introns – which diverge in their consensus sequences at the 5′ splice site, branch point sequence and 3′ splice site (Turunen et al., 2013). The vast majority of introns are major introns (>99%), whereas a small minority (<1%) represents minor introns. Specifically, 722 minor introns are found in only 699 human genes (Olthof et al., 2019), and thus, most minor intron-containing gene (MIGs) contain a single minor intron, flanked by ‘normal’ major introns. The existence of major and minor introns, with different splicing consensus sequences, requires not one, but two different splicing machineries, the so-called major ‘U2-dependent’ and minor ‘U12-dependent’ spliceosomes, each containing their own unique core components.

Minor introns and the minor spliceosome are highly conserved; in fact, the position of minor introns within genes is more conserved than that of major introns (Basu et al., 2008). Minor introns, which can be traced back to the last eukaryotic common ancestor, are enriched in numerous gene families, some of which are crucial for cardiomyocyte function, such as voltage-gated Na^+^ and Ca^2+^ channels, and mitogen activated-protein kinases (MAPKs) (Baumgartner et al., 2019). Although the exact function of minor introns has remained an enigma, the fact that they have endured in modern genomes indicates that they confer an evolutionary advantage, despite the increased metabolic load on the cell of maintaining two parallel splicing machineries (Baumgartner et al., 2019). Recent studies have uncovered a crucial functional difference between minor and major splicing; minor introns generally splice at a slower rate than major introns (Patel et al., 2002). As a consequence, minor introns are on average 2-fold more retained than their neighbouring major introns (Niemelä et al., 2014; Younis et al., 2013). It has been proposed that unspliced transcripts are either trapped in the nucleus, or degraded by the exosome or via nonsense-mediated decay (NMD) (Patel et al., 2002). This suggests that minor introns act as regulatory switches to control the expression of sets of MIGs, which can be accomplished by altering the levels of minor spliceosome components. Indeed, Younis et al. demonstrated that *U6atac*, one of the small nuclear (sn)RNAs specific to the minor spliceosome, has a short half-life and can be rate limiting for minor intron splicing in HeLa cells. They showed that *U6atac* can be stabilized by activated p38 MAPKs, which is produced in response to cellular stress. This resulted in enhanced splicing of minor introns and increased expression of numerous genes that contain them. It was concluded that the minor spliceosome enables proteins to be produced rapidly in response to stress, bypassing the need for a new round of transcription (Younis et al., 2013).

Mutations in core components of the minor spliceosome are responsible for several complex syndromes. To date five minor spliceosome congenital human diseases have been described: isolated growth hormone deficiency (IGHD), microcephalic osteodysplastic primordial dwarfism type I/Taybi–Linder syndrome (MOPD1/TALS), Roifman syndrome (RFMN), Lowry Wood syndrome (LWS) and early onset cerebellar ataxia (EOCA) (Verma et al., 2018). These pathologies show diverse and pleiotropic symptoms, but all share a neurological component with varying degrees of severity. Although these diseases mostly underscore the importance of the minor spliceosome in neurons, its function in the cardiovascular system has, to the best of our knowledge, not yet been explored. Nevertheless, the fact that numerous MIGs are known to encode protein families essential for cardiomyocyte biology (e.g. Na^+^ and Ca^2+^ channels, MAPKs and calpains), raises the intriguing possibility that minor spliceosome regulation is critical for cardiac (electro-)physiology.

Here, we investigated whether minor intron splicing may constitute a regulatory mechanism to control gene expression in the heart. By knocking down the minor spliceosome component *U6atac* in neonatal rat ventricular myocytes (NRVMs), we demonstrate robust effects on minor intron retention in the Na^+^ channel *Scn5a* (encoding Na_v_1.5) and the L-type Ca^2+^ channel *Cacna1c* (encoding Ca_v_1.2), and show the functional consequences through patch-clamp analysis.

Altogether, our study provides the first evidence that alterations in minor splicing activity modulate cardiac electrophysiology, which may be of vital importance in genetic diseases affecting minor spliceosome components and in acquired diseases in which minor spliceosome components are dysregulated, including heart failure.

## RESULTS

### Minor-intron containing genes are expressed in the heart

**Fig. 1.**
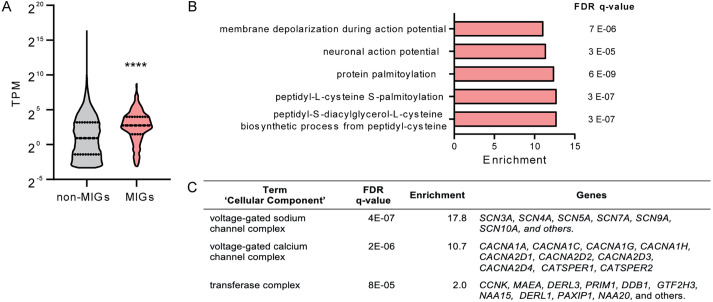
**Minor-intron-containing genes expressed in the heart.** (A) Expression of MIGs and genes without minor introns in non-diseased hearts from data previously published in Heinig et al. (2017) (*n*=100). Data is presented as a violin plot with median and quartiles marked. ****P<0.0001 (Mann–Whitney test). TPM, transcripts per million mapped reads. (B) ‘Biological process’ and (C) ‘cellular component’ terms enriched in the subset of MIGs expressed in the human heart according to gene ontology enrichment analysis (GOrilla). The FDR q-value is the adjusted *P*-value for multiple testing using the Benjamini and Hochberg method. Enrichment is the percentage of genes in a specific gene ontology term in the input (MIGs) divided by the percentage in the background (all genes).

To explore the potential relevance of minor intron splicing in cardiac physiology, we first examined the expression of minor-intron containing genes (MIGs) in published RNA sequencing data from 100 human non-diseased hearts (left ventricular tissue) (Heinig et al., 2017). Of the 699 previously identified MIGs (Olthof et al., 2019), we found a total of 582 expressed in these hearts (Table S6). The average expression of MIGs was significantly higher than the expression of genes without minor introns (Fig. 1A), indicating that MIGs constitute an actively expressed subset of genes in the heart.

As an initial characterization of MIGs in the heart, we performed gene ontology enrichment analysis using the GOrilla bioinformatic tool (Eden et al., 2009). As shown in Fig. 1B, MIGs expressed in the heart are enriched in ‘biological process’ terms related to electrophysiology and to protein palmitoylation. Analysis of the ‘cellular component’ terms revealed an enrichment in the categories of ‘voltage gated Na^+^ channel complex’ and ‘voltage gated Ca^2+^ channel complex’ (Fig. 1C), which includes the gene families encoding the α-subunit of voltage gated Na^+^ channels (e.g. *SCN5A* and *SCN10A*) and numerous subunits of the voltage-gated Ca^2+^ channels (e.g. *CACNA1C* and *CACNA1G*).

### Knockdown of *U6atac* results in minor intron retention in NRVMs

**Fig. 2.**
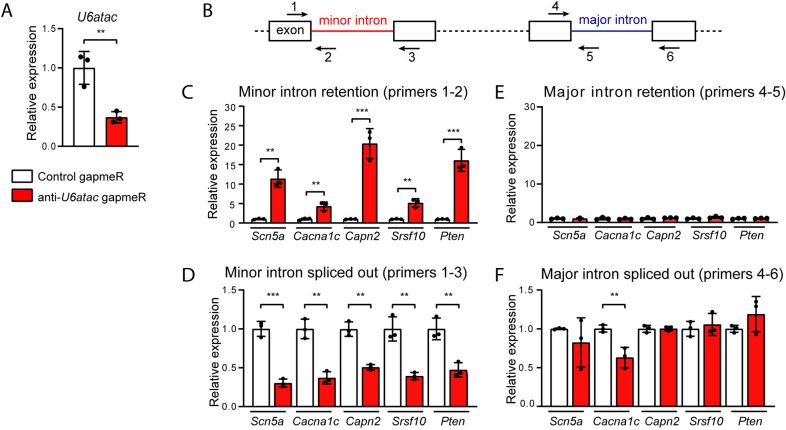
**Effect of *U6atac* knockdown on minor intron retention in NRVMs.** (A) Relative expression of the minor splicing component *U6atac* 48 h after gapmeR-mediated knockdown in NRVMs. (B) Schematic illustration of the primer design for qPCR analysis. (C–F) Relative expression of minor introns (C), minor intron-flanking exons (D), major introns (E) and major intron-flanking exons (F) as detected by qPCR using primers depicted in B. Relative expression was calculated using as reference the geometric mean of *Gapdh*, *Hprt1* and *Eef1e1*. Data are presented as mean±s.d. for 3 wells per condition. **P<0.01; ***P<0.001 (unpaired two-tailed Student's *t*-test).

Given our observation that numerous MIGs are expressed in the heart and that minor introns are enriched in, for example, voltage-gated Na^+^ and Ca^2+^ channels, we next investigated the impact of minor splicing on MIG expression in cardiomyocytes. To this end, we performed a gapmeR-mediated knockdown of the non-coding snRNA *U6atac* in NRVMs (Fig. 2A). We chose to knockdown *U6atac* because it is an essential component of the minor spliceosome that is known to be a limiting factor in minor splicing (Younis et al., 2013). We transfected six-well plates with NRVMs with either an anti-*U6atac* or a non-targeting negative control gapmeR. The anti-*U6atac* gapmeR successfully knocked down *U6atac* up to 60% at 48 h after transfection (Fig. 2A). To quantify minor retention in these transfected cells, we designed four sets of qPCR primers surrounding the minor and one of the major introns for a panel of five highly expressed MIGs in cardiomyocytes: the Na^+^ channel *Scn5a*, the Ca^2+^ channel *Cacna1c*, the Ca^2+^-activated protease *Capn2*, the phosphatase *Pten* and the splicing factor *Srsf10*. As depicted in Fig. 2B, the quantitative (q)PCR analysis included a primer set detecting the retained minor intron (primer 1 and 2), a primer set that detects the correctly spliced exons surrounding the minor intron (primer 1 and 3), a primer set that detects intron retention of an unrelated major intron of the same gene (primer 4 and 5) and a primer set that detects the transcript in which that major intron is spliced out (primer 4 and 6). The latter primer set also provides an indication of the expression level of the transcript. Overall, with this strategy, we were able to discriminate between the fully spliced transcripts and the transcripts that still contained the retained intron. The major introns were included to confirm that major intron retention was not affected by our approach (Fig. 2B).

Knockdown of *U6atac* led to a strong increase in retention of all the minor introns tested: intron 3 of *Scn5a*, intron 13 of *Cacna1c*, intron 14 of *Capn2*, intron 1 of *Pten* and intron 2 of *Srsf10* (Fig. 2C). Accordingly, levels of the corresponding minor intron-flanking exons spliced together decreased (Fig. 2D), which, as expected, shows that minor intron retention occurs at the expense of proper splicing. Interestingly, a 60% reduction in *U6atac* produced a similar reduction (i.e. ∼50–80%) in minor intron splicing. This ≈1:1 reduction ratio in *U6atac* and properly spliced transcripts underscores that *U6atac* is an essential component of the minor spliceosome. We did not observe an increase in major intron retention, which further validates that the knockdown of *U6atac* affects only minor and not major splicing (Fig. 2E). The mRNA expression levels of the MIGs was generally unaltered after *U6atac* knockdown (Fig. 2F), with the exception of *Cacna1c*, which was downregulated.

To validate the specificity of our approach, we repeated the experiment with a different anti-*U6atac* gapmeR (Fig. S1A–C) and with gapmeRs against the minor spliceosome component *U4atac* (Fig. S1D,E). With the four different gapmeRs, we were able to induce minor intron retention, which indicates that the levels of both *U4atac* and *U6atac* are essential for proper minor splicing activity.

NMD may eliminate transcripts in which the minor intron is retained. To test whether this is indeed the case after *U6atac* knockdown, we inhibited the NMD pathway 24 h after gapmeR transfection by incubating the NRVMs with the translation inhibitors cycloheximide or emetine for 3 h. As shown in Fig. S2, cycloheximide or emetine did not further increase minor intron retention in *Scn5a* or *Cacna1c* or mRNA levels, indicating that these transcripts with minor introns retained are not targeted by NMD. We included *Myh7b* – a bona fide target of the NMD pathway in cardiomyocytes – as a positive control for NMD inactivation (Bell et al., 2010).

### Impact of minor splicing knockdown on cardiomyocyte morphology

**Fig. 3.**
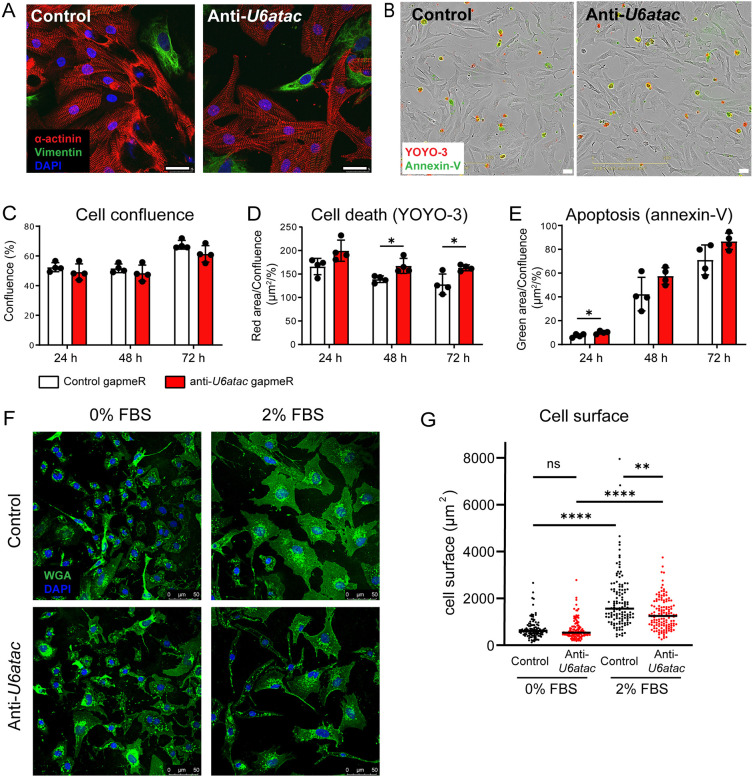
**Impact of minor splicing knock-down on cardiomyocyte growth, survival and size.** (A) Representative immunocytochemistry of three experiments of NRVMs at 48 h after transfection with control or anti-*U6atac* gapmeRs. Red, α-actinin (cardiomyocyte marker); green, vimentin (fibroblast marker); blue, DAPI. (B) Representative phase-contrast images of NRVMs and (C) quantification of cell confluence, (D) cell death and (E) apoptosis. Data are presented as mean±s.d. for 4 wells per condition. *P<0.05 (unpaired two-tailed Student's *t*-test). (F) Representative images of transfected NRVMs after 48 h incubation in medium with 0% FBS or 2% FBS and (G) quantification. Green, WGA (membrane marker); blue, DAPI. *n*>100 cells per condition. **P<0.01; ****P<0.0001; ns, not significant [one-way ANOVA on ranks (Kruskal–Wallis test), followed by Dunn's test for post hoc analyses]. Scale bars: 25 µm (A,B); 50 µm (F).

To get a global impression of the morphology and condition of the cells after disrupting the minor spliceosome, we performed immunostainings 48 h after anti-*U6atac* and control gapmeR transfections. Immunocytochemistry with an α-actinin antibody after *U6atac* knockdown did not reveal obvious differences in the sarcomere structure of NRVMs, nor did we observe evident changes in the morphology or numbers of residual fibroblasts as detected by vimentin staining (Fig. 3A). Using automated image acquisition (Incucyte; Fig. 3B), we performed live-cell analysis at three time points (24 h, 48 h and 72 h) after transfection of the gapmeRs. As shown in Fig. 3C, there was no difference in cell confluence over time between anti-*U6atac* and control gapmeRs. To analyze cell death, we employed a fluorescent probe based on Annexin V (Annexin V-ATTO-488), to measure phosphatidylserine exposure during apoptosis, and the DNA dye YOYO-3, to measure permeabilized dead cells. The fluorescent areas of the Annexin-V probe and YOYO-3 in each image was analyzed to calculate the ratio of apoptotic and dead cells relative to the total cell area. Cell death remained rather constant over time, with a slight, but significant increase in the anti-*U6atac* condition at 48 h and 72 h after transfections (Fig. 3D). Apoptosis was significantly increased at the 24 h time point after *U6atac* knockdown, but not at later timepoints (Fig. 3E). Given that cell confluence was not decreased in the *U6atac* knockdown groups over time, we conclude that inhibition of minor intron splicing does not affect cell viability or lead to evident damage of the cells.

We examined the hypertrophic response of the NRVMs after *U6atac* knockdown by culturing cells in serum-free medium or in medium with 2% fetal bovine serum (FBS) for 48 h (Fig. 3F,G). We quantified cell surface areas after staining the cell membrane with wheat germ agglutin (WGA)-488, as a measure of hypertrophy. Without serum (0% FBS), we did not observe a difference in cell size after *U6atac* knockdown. Serum stimulation (2% FBS) induced hypertrophy in both gapmeR groups; however, the hypertrophic response was reduced in the anti-*U6atac* group. These results indicate that *U6atac* knockdown also affects cardiomyocyte hypertrophy, which may be mediated by PTEN, a key regulator in phosphoinositide 3-kinase (PI3K)/AKT signaling, or other MIGs that participate in the hypertrophic response.

### Minor intron retention of *Scn5a* decreases Na_v_1.5 expression and Na^+^ current density

Given that our pathway analysis revealed that cardiac MIGs are enriched for ion channels, this points to a potential link between minor intron splicing and electrophysiological properties of the cardiomyocyte. With minor introns being enriched in both voltage-gated Ca^2+^ and Na^+^ channels genes, we hypothesized that the expression of these ion channels proteins critically depends on the activity of the minor spliceosome. We specifically focused on the predominant voltage-dependent Na^+^ and Ca^2+^ α-subunits expressed in the heart, *Scn5a* (encoding Na_v_1.5) and *Cacna1c* (encoding Ca_v_1.2).

**Fig. 4.**
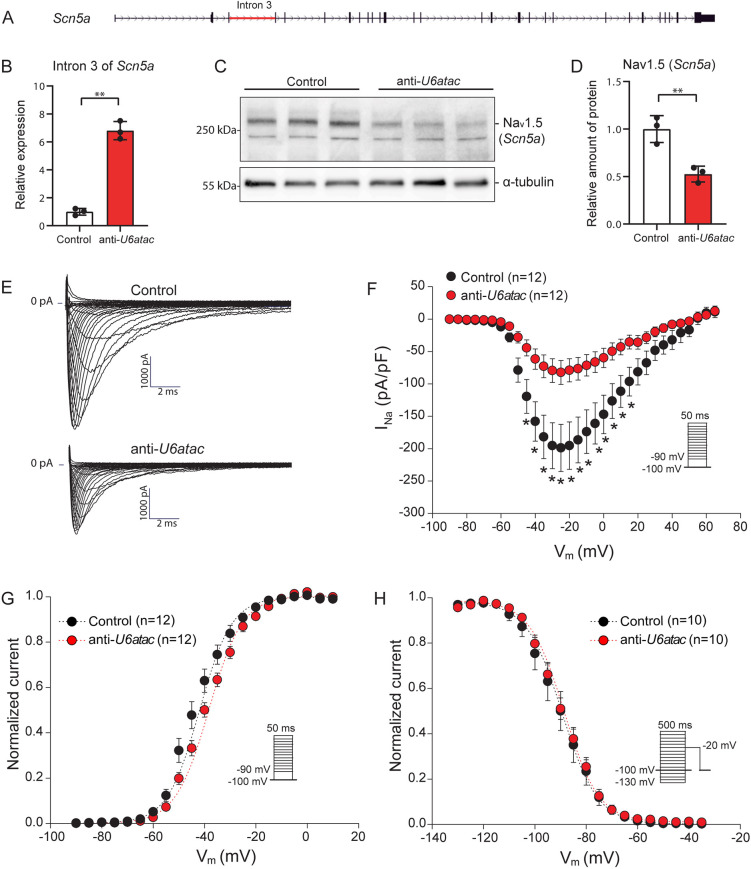
**Minor intron retention of *Scn5a* reduces translation into Na_v_1.5 and *I*_Na_ density**. (A) Gene structure of *Scn5a* with its minor intron indicated in red. (B) Minor intron retention in *Scn5a* 48 h after *U6atac* knockdown measured by qRT-PCR. (C) Na_v_1.5 (*Scn5a*) detection by western blot and (D) its quantification. Data in B,D are presented as mean±s.d., 3 wells per condition. **P<0.01 (unpaired two-tailed Student's *t*-test). (E) Representative *I*_Na_ traces recorded from NRVMs transfected with control or anti-*U6atac* gapmeR. (F–H) Average *I*_Na_–voltage relationships (F), *I*_Na_–voltage dependence of activation (G) and inactivation (H). Insets show voltage protocols. Patch-clamp data are presented as mean±s.e.m.; *n* indicates number of cardiomyocytes. *P<0.05 (two-way repeated measures ANOVA followed by Holm–Sidak test for post hoc analyses).

Consistent with the experiment shown in Fig. 2C, *U6atac* knockdown in NRVMs resulted in strong retention of the minor intron of *Scn5a* (Fig. 4A,B). In turn, this decreased the levels of the Na_v_1.5 protein, as shown by western blotting (Fig. 4C,D), while leaving the mRNA levels unaffected (Fig. 2F). To assess the functional consequences of these molecular alterations, we studied the Na^+^ current (*I*_Na_) characteristics in anti-*U6atac*- and control-treated NRVMs using conventional patch-clamp. A fluorescently labeled gapmeR was co-transfected along with the anti-*U6atac* and control gapmeRs to measure transfected cells only. Fig. 4E illustrates representative *I*_Na_ traces induced by depolarizing steps from a holding potential of −100 mV 48 h after transfection with the gapmeRs. The *I*_Na_–voltage relationship (Fig. 4F) showed a strong and significant decrease in the *I*_Na_ density after *U6atac* knockdown, reaching a ∼59% reduction at −25 mV (−82.3±18.5 pA/pF in *U6atac* versus −198.8±36.4 pA/pF in control cells; *P*<0.05; Table S4). Comparison of activation and inactivation parameters [the half-voltage of (in)activation *V*_1/2_ and the slope factor *k*] revealed a significant shift of *V*_1/2_ of activation towards a more positive potential (+4.4 mV) and a decrease in the activation curve steepness (increased *k*) after *U6atac* knockdown (Fig. 4G; Table S4). These changes in kinetic properties can lead to a decrease in *I*_Na_ in response to channel voltage activation. In contrast, *I*_Na_ inactivation parameters remained unaltered (Fig. 4H; Table S4). Hence, a decrease in minor spliceosome activity can affect Na_v_1.5 protein levels and consequently reduce *I*_Na_ density in cardiomyocytes.

### Minor intron retention of *Cacna1c* decreases Ca_v_1.2 expression and L-type Ca^2+^ current

**Fig. 5.**
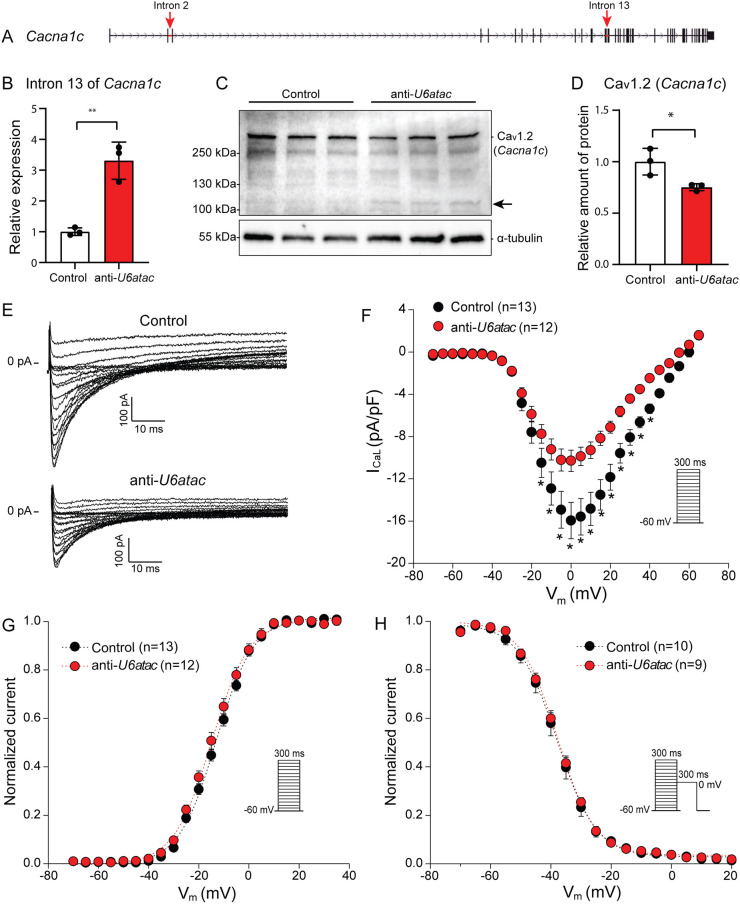
**Minor intron retention of *Cacna1c* hampers translation into Ca_v_1.2 and reduces *I*_CaL_ density.** (A) Gene structure of *Cacna1c* with minor introns indicated with red arrows. (B) Minor intron retention in *Cacna1c* after *U6atac* knockdown measured by qRT-PCR. (C) Ca_v_1.2 (*Cacna1c*) detection by western blot and (D) its quantification. Black arrow indicates a potential truncated isoform of Ca_v_1.2. Data in B,D are presented as mean±s.d., 3 wells per condition. *P<0.05, **P<0.01 (unpaired two-tailed Student's *t*-test). (E) Representative *I*_CaL_ traces recorded from NRVMs co-transfected with control or anti-*U6atac* gapmeR. (F–H) Average *I*_CaL_–voltage relationships (F), *I*_CaL_-voltage dependence of activation (G) and inactivation (H). Insets show voltage protocols. Patch-clamp data are presented as mean±s.e.m.; *n* indicates number of cardiomyocytes. *P<0.05 (two-way repeated measures ANOVA followed by Holm–Sidak test for post hoc analyses).

*Cacna1c*, which encodes the L-type Ca^2+^ channel Ca_v_1.2, contains 44 introns, two of which are minor introns, namely, intron 2 and 13 (Fig. 5A; Fig. S3). Minor intron retention of *Cacna1c* after U6atac knockdown (Fig. 5B) resulted in a significant decrease of Ca_v_1.2 protein levels (∼30%) as assessed by western blotting (Fig. 5C,D). In addition to this decrease, a new band of smaller molecular mass (≈100 kDa) appeared upon *U6atac* knockdown (Fig. 5C). Interestingly, this smaller band does not correspond to the size of the predicted alternative open reading frame that arises when the minor intron is retained in *Cacna1c* (Fig. S3). It is therefore not clear whether the 100 kDa band resulted from an aberrantly spliced isoform or whether it represents a degraded or cleaved product.

To assess the functional consequence of the decreased Ca_v_1.2 expression, we performed patch-clamp analysis to investigate L-type Ca^2+^ current (*I*_CaL_). Representative *I*_CaL_ traces recorded from NRVMs transfected with the control or anti-*U6atac* gapmeR are depicted in Fig. 5E. The current-voltage (*I*–*V*) relationship (Fig. 5F) showed a robust and significant decrease in the *I*_CaL_ density after *U6atac* knockdown, reaching a ∼36% reduction at −0 mV (control −16.0±1.7 pA/pF versus anti-*U6atac* −10.3±1.0 pA/pF, *P*<0.05; Table S5). Finally, the *I*_CaL_ voltage dependence of activation and inactivation parameters (*V*_1/2_ and *k*) was similar in both groups (Fig. 5G,H; Table S5). Taken together, these data demonstrate that a decrease in minor spliceosome activity leads to a reduction in Ca_v_1.2 protein levels and consequently to a reduced I_CaL_ in cardiomyocytes.

### Dysregulation of minor spliceosome components in human heart disease

**Fig. 6.**
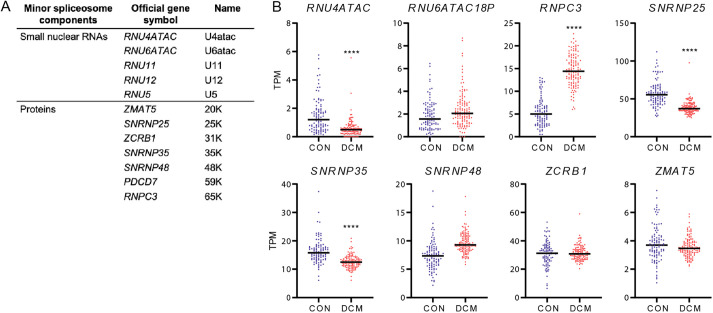
**Minor spliceosome components expressed in the human failing heart.** (A) Minor U12-dependent spliceosome components and (B) its expression in 100 non-diseased (CON) and 128 dilated cardiomyopathy (DCM) hearts. **** adjusted *P*<0.0001.

The minor spliceosome comprises five snRNAs (*U11*, *U12*, *U4atac*, *U5* and *U6atac*) and seven associated proteins [encoded by *ZMAT5* (20K), *SNRNP25* (25K), *ZCRB1* (31K), *SNRNP35* (35K), *SNRNP48* (48K), *PDCD7* (59K) and *RNPC3* (65K)] (Fig. 6A). These components are exclusive to the minor spliceosome and are not found in the major spliceosome, except for the snRNA *U5*, which is common to both spliceosomes (Will et al., 2004). To investigate whether human heart disease might be associated with perturbations in the activity of the minor spliceosome, we compared the expression of these components in the 100 non-diseased hearts with that of 128 dilated cardiomyopathy (DCM) hearts (Heinig et al., 2017). Interestingly, we found that a number of these minor spliceosome components were differentially expressed in DCM (Fig. 6B; Fig. S4). As shown in Fig. 6B, some components were significantly downregulated (i.e. *RNU4ATAC*, *SNRNP25* and *SNRNP35*) whereas others were upregulated (i.e. *RNPC3*). In addition, we also plotted the expression of major spliceosome components in this dataset (Fig. S4). This shows that some major spliceosome components are dysregulated in DCM as well. This is in line with previous studies reporting decreased splicing efficiency in heart failure (Kong et al., 2010), but it also indicates that dysregulation of minor spliceosome components is not a unique feature in DCM.

Transcriptional dysregulation of minor spliceosome components did not lead to overt and systematic changes in mRNA levels of MIG in this dataset, although some MIGs were found to be differentially expressed in DCM hearts (e.g. *STX10*, *RABGGTA* and *C2CD3*) (Fig. S5). Nevertheless, it is conceivable that perturbations in minor spliceosome activity may have affected protein levels of MIGs through intron retention, without decreasing mRNA levels.

## DISCUSSION

In this study, we show that a large proportion (582/699) of minor intron-containing genes (MIGs) is expressed in the human heart and that a substantial number of these genes are directly involved in cardiac electrophysiology (e.g. *SCN5A* and *CACNA1C*). To explore a potential role of the minor spliceosome in the electrical activity of cardiomyocytes, we knocked down the minor spliceosome-specific component *U6atac* in NRVMs, and observed that loss of *U6atac* hampered splicing of the minor introns of *Scn5a* and *Cacna1c* and reduced their protein levels. Consequently, reduced levels of these voltage-gated ion channels decreased *I*_Na_ and *I*_CaL_, as demonstrated by patch-clamp analysis. Together, our findings identify a novel mechanism by which *I*_Na_ and I_CaL_ might be modulated in cardiac pathologies in which the expression of components of the minor spliceosome is dysregulated.

Na^+^ channels play an essential role in the initiation and propagation of the cardiac action potential by allowing the Na^+^ influx during the depolarizing phase of the action potential (Remme and Wilde, 2014). The α-subunit of the Na^+^ channel contains essential elements for its function, including the ion-conducting pore. Nine different α-subunits of the Na^+^ channel (encoded by *SCN1A* through *SCN11A*) are expressed throughout the body and each and every one of these α-subunits contains a minor intron (Baumgartner et al., 2019; Patel and Steitz, 2003; Wu and Krainer, 1999). Na_v_1.5, encoded by the *SCN5A* gene, is the predominant α-subunit in the heart. Alterations in Na_v_1.5 and reduced Na^+^ current density have been reported in common pathophysiological conditions such as ischemia and heart failure (Rook et al., 2012). Na^+^ current reduction slows cardiac conduction, and as such might contribute to the increased arrhythmic risk in the setting of these disorders (Remme and Wilde, 2014). The underlying molecular mechanism(s) could relate to regulation at the (post)transcriptional level, trafficking of Na_v_1.5 to the cell membrane and/or post-translational modifications of Na_v_1.5 (phosphorylation, glycosylation, ubiquitylation, etc.). An example of regulation at the post-transcriptional level is the abnormal C-terminal splicing of *SCN5A* that occurs in heart failure, which produces truncated and non-functional Na_v_1.5 channels (Shang et al., 2007). In our current study, we raise the intriguing possibility that changes in minor spliceosome activity may also contribute to changes at the post-transcriptional level, resulting in minor intron retention and reduced Na^+^ current density in heart failure. In a large RNA-seq database of 100 non-diseased and 128 failing hearts (i.e. DCM), we found that several minor spliceosome components are differentially expressed. Since some components were significantly downregulated (i.e. *RNU4ATAC*, *SNRNP25*, *SNRNP35*), whereas others were upregulated (i.e. *RNPC3*) in DCM, it is difficult to predict whether the downstream effects would either represent an increase or decrease in minor intron retention. In this same RNA-seq dataset, we did not detect clear and global differences in the mRNA expression of MIGs. However, this is not unexpected, as it is conceivable that protein expression of MIGs was affected without reducing mRNA levels. In fact, for four out of five MIGs tested after *U6atac* knockdown, we indeed observed an increase in minor intron retention without reducing mRNA levels (Fig. 2F). Furthermore, inhibition of minor spliceosome activity by *U6atac* knockdown did show a reduction in Na_v_1.5 protein by western blotting with a concomitant decrease in Na^+^ current density, whereas it left *Scn5a* mRNA levels unaffected. Future studies investigating the relationship between minor spliceosome activity and Na^+^ channel function in the hearts of knockout mice or human patients with specific minor spliceosome mutations will be awaited with great interest.

Minor introns are also present in voltage-gated Ca^2+^ channels. These channels open at depolarizing membrane potentials leading to an influx of Ca^2+^ into the cell. The entry of Ca^2+^ constitutes an intracellular signal that triggers a wide spectrum of Ca^2+^-sensitive mechanisms, such as Ca^2+^-induced Ca^2+^ release and the sarcomere contraction in cardiomyocytes (Bodi et al., 2005). Ca^2+^ channels are composed of five different subunits: α1, α2, δ, β and γ. The α1 subunit is the core component of the channel containing the conduction pore, the voltage-sensing domain and the gating apparatus. The other four auxiliary subunits have regulating functions (Bodi et al., 2005). The heart expresses two types of Ca^2+^ channels that differ in their α1 subunit; L-type channels, which open at high voltages are mostly expressed in ventricular cardiomyocytes, and T-type channels, which open at low voltages, are only present in pacemaker, atrial and Purkinje cells. There is at least one minor intron in all 10 homologous genes encoding α1 subunits (*CACNA1A* to *CACNA1S*) and in the four different α2δ genes encoding the α2 and δ subunits genes (*CACNA2D1* to *CACNA2D4*) (Baumgartner et al., 2019; Wu and Krainer, 1999). Our current findings demonstrate that a decrease in minor spliceosome activity induced by *U6atac* knockdown resulted in a reduction of *Cacna1c* mRNA, Ca_v_1.2 protein and *I*_CaL_. Interestingly, *U6atac* knockdown also yielded a truncated isoform of the Ca_v_1.2 protein of ∼100 kDa. Whether this truncated variant represents an alternative splice isoform of *Cacna1c*, triggered by activation of cryptic splice sites when the minor intron is retained is currently unknown. It is also not known whether this truncated Ca_v_1.2 variant is expressed in (patho)physiological conditions of the heart, which exons it comprises and whether it has functional relevance. That splice variants in *Cacna1c* are able to affect Ca^2+^ currents was recently demonstrated in an elegant study by Li et al., who showed that exclusion of alternative exon 33 in *Cacna1c* leads to greater *I*_CaL_ density. A mouse model in which exon 33 was deleted revealed a proarrhythmogenic phenotype, including premature ventricular contractions, tachycardia and lengthened QT interval (Li et al., 2017; Liao et al., 2009). Complementary to such alternative exon usage, our findings point to an independent means by which intron retention regulates levels of the encoded proteins.

In our studies, we made use of immature NRVMs instead of adult cardiomyocytes because they are suitable for transfections and for prolonged culturing. There are differences between neonatal and adult cardiomyocytes in terms of electrophysiology, but we expect that inhibition of minor splicing also affects Na^+^ and L-type Ca^2+^ currents in fully mature cardiomyocytes. During cardiomyocyte maturation, alternative splicing of exon 6 of *Scn5a* (Onkal et al., 2008) and exons 21, 31, 32 and 33 of *Cacna1c* (Diebold et al., 1992; Hu et al., 2016; Liao et al., 2015) is known to occur, and is associated with differences in Na^+^ and L-type Ca^2+^ current properties. However, these alternative splice events occur downstream of the minor introns of *Scn5a* and *Cacna1c* and should be present in fetal and mature isoforms of both genes.

So far, no cardiac disease has been linked directly to minor intron splicing. Mutations causing loss-of-function of the minor spliceosome are responsible for rare and complex syndromes, mainly affecting growth and the nervous system (Benoit-Pilven et al., 2020; Doggett et al., 2018; Merico et al., 2015; Verma et al., 2018). However, some MOPD1/TALS and Roifman syndrome patients (caused by mutations in *RNU4ATAC*) present with cardiac defects, including coarctation of aorta, Tetralogy of Fallot, atrial septal defect, ventricular septal defect and non-compaction of the heart. This suggests that a deficiency in minor splicing is detrimental for cardiac development (Edery et al., 2011; Merico et al., 2015). It is currently unknown whether patients with loss-of-function mutations of the minor spliceosome also present with electrical disorders or arrhythmias.

In mice, constitutive deletion of the minor spliceosome components *Rnu11* or *Rnpc3* results in early embryonic lethality (Baumgartner et al., 2018; Doggett et al., 2018). Using conditional knockout mouse models, Baumgartner et al. were recently able to show that deletion of *Rnu11* specifically in the embryonic mouse cortex resulted in microcephaly due to increased abnormal intron retention of MIGs involved in the cell cycle, DNA damage control and apoptosis (Baumgartner et al., 2018). Furthermore, constitutive deletion of *Rnpc3* in adult mice resulted in reduced levels of lymphocytes, monocytes, erythrocytes and thrombocytes, decreased thymus size and degeneration of lining of the gastrointestinal tract within 1 week after deleting *Rnpc3* (Doggett et al., 2018). These conditional knockout models indicate that proper MIG expression is required for the survival of rapidly dividing cell population and that differentiated cells are less vulnerable to minor spliceosome inactivation (Baumgartner et al., 2019). Unfortunately, the hearts of *Rnpc3*- and *Rnu11*-knockout mice have not been subjected to careful functional and biochemical analyses. Our study indicates that it is likely that the hearts of these mice are compromised on the (electro)physiological level, due to the reduced expression and function of, for example, *Scn5a* and *Cacna1c*.

There is evidence that some accessory splicing factors regulating minor splicing are important for a normal function of the heart. For example, SMN1 has recently been identified as a splicing factor involved in minor splicing (Boulisfane et al., 2011; Li et al., 2020; Lotti et al., 2012). SMN1 is required for the survival of motor neurons, and mutations in this gene are responsible for spinal muscular atrophy. Spinal muscular atrophy patients also present with a cardiac phenotype, which includes bradycardia and premature heart failure; symptoms that were initially thought to originate from innervation problems. However, cell models have shown that SMN1 is also important for proper cardiomyocyte function (Khayrullina et al., 2020). Another potential minor splicing regulator in the heart is the ubiquitously expressed RNA-binding protein FUS. Mutations in FUS are responsible for amyotrophic lateral sclerosis (ALS). The same mutations causing ALS also affect minor intron retention, and might contribute to the arrhythmias and sudden cardiac death observed in ALS patients (Reber et al., 2016).

More research is needed to address a possible contribution of aberrant minor intron splicing in human cardiac disease and to evaluate cardiac function in minor spliceosome diseases. We interrogated the expression of MIGs and minor spliceosome components in the hearts of DCM patients; however, we were not able to investigate minor intron retention levels, as this requires ultra-deep high-throughput RNA sequencing coupled with advanced bioinformatic algorithms, which requires experiments beyond the scope of this study. Besides DCM and heart failure, it will be interesting to investigate a possible contributing role for minor intron retention in (inherited) cardiac disorders in which the electrical activity of the heart is affected, such as for instance Brugada syndrome (which is associated with mutations in *SCN5A* and *CACNA1C*), and arrhythmogenic cardiomyopathy, disorders with an increasingly recognized complex genetic basis (Cerrone et al., 2019).

In conclusion, we here provide the first evidence that alterations in minor splicing can affect cardiac electrophysiology by modulating the expression and function of voltage-gated ion channels in cardiomyocytes. This novel insight may be of vital importance in genetic disorders affecting minor spliceosome components and in acquired diseases in which minor spliceosome components are dysregulated, including heart failure.

## MATERIALS AND METHODS

### RNA-seq data and differential gene expression analysis

The paired-end reads from 100 human non-diseased and 128 dilated cardiomyopathy (DCM) hearts (left ventricle tissue) previously generated (Heinig et al., 2017) were aligned against the human genome reference hg38 using STAR2 (Dobin et al., 2013) (Version 2.7) and the Ensemble GRCh38 release 100 annotation file. Differential gene expression analysis was performed using the R Bioconductor package, DESeq2 (Love et al., 2014) (Bioconductor release 3.12). Genes with transcripts per million (TPM)≥0.1, absolute log_2_ fold change ≥0.58 and adjusted *P*-value cutoff ≤0.05 were deemed significantly differentially expressed.

### Intron location and pathway analysis

Minor intron coordinates were obtained from Minor Intron Database (MIDB, https://midb.pnb.uconn.edu/; Olthof et al., 2019). Minor introns in rat were selected by means of homology with the human sequences.

Gene ontology enrichment analysis was performed using the online tool Gorilla (Eden et al., 2009). Enrichment analysis was performed on MIGs with a mean TPM value ≥0.1 against all the genes expressed above the same threshold in human non-diseased hearts. Gene ontology terms with FDR q-value≤0.001 were considered significant.

### NRVM isolation and gapmeR transfection

Animal studies were approved by the Institutional Animal Care and Use Committee of the University of Amsterdam and carried out in compliance with the guidelines of this institution and the Directive 2010/63/EU of the European Parliament.

NRVMs from 0–2-day-old Wistar rats (Janvier Labs, Le Genest-Saint-Isle, France) were isolated as described previously (den Haan et al., 2013). In short, pups were anesthetized by isoflurane and hearts were excised after decapitation. Ventricles were cut into small pieces, which were incubated overnight in a rotating platform at 4°C in Hank's balanced salt solution (HBSS; Gibco, Ref 14170-088, Paisley, UK) containing 1 mg/ml trypsin (USB, Ref 22720, Cleveland, OH, USA). The next day, cells were dissociated using 1 mg/ml collagenase type 2 (Worthington, Ref LS004177, Lakewood, NJ, USA) in HBSS. Cells were collected and resuspended in TUNG medium [M199 culture medium (Gibco, Ref 31150-022), 1% HEPES (Gibco, Ref 15630-080), 1% non-essential amino acids (NEAA; Gibco, Ref 11140-050), 2 mg/l vitamin B12 (Sigma, Ref V2876, St Louis, MO, USA), 3.5 g/l glucose, 1% penicillin-streptomycin (Gibco, Ref 15140-122)] supplemented with 10% FBS (Biowest, Ref S1810-500, Riverside, MO, USA). The cell suspension was pre-plated to separate myocytes from fibroblasts. After 2 h, non-adhered myocytes were collected and counted with a LUNA™ Automated Cell Counter (Logos Biosystems, Anyang, South Korea).

10^6^ NRVMs were plated on six-well plates coated with fibronectin (Corning, Ref 356008, Bedford, MA, USA). At 4–20 h after seeding, NRVMs were transfected with 25 nM control or anti-*U6atac* gapmeRs (Qiagen, Ref 339511, Germantown, MD, USA) using Lipofectamine 2000 (Thermo Fisher Scientific, Ref 11668-019, Carlsbad, CA, USA) in TUNG medium without FBS. After 6 h of incubation, medium was replaced by TUNG with 2% FBS. Cells were harvested or analyzed at 48 h after transfection. Experiments were repeated at least in three different isolations, plating three wells per condition each time. GapmeR sequences are found in Table S1.

For NMD inhibition, NRVMs were incubated with the translation inhibitors cycloheximide (300 µg/µl; Sigma, Ref 01810) or emetine (150 µg/µl; Sigma, Ref E2375) for 3 h at 24 h after gapmeR transfection.

For patch-clamp experiments, 10^5^ NRVMs were seeded over fibronectin-coated glass coverslips on 24-well plates and co-transfected with 25 nM gapmeRs and 25 nm FAM-labeled control (Qiagen, Ref 339515) using Lipofectamine 2000. At 48 h after transfection, Na^+^ and Ca^2+^ current recordings were performed in fluorescent cardiomyocytes with the use of the patch-clamp technique.

### RNA isolation and qRT-PCR

RNA was extracted using TRI reagent (Sigma, Ref T9424) following manufacturer's instructions. 500 ng of total RNA was treated with DNase I (Invitrogen, Ref 18068-015, Waltham, MA, USA) and retrotranscribed into cDNA with random hexamers (Invitrogen, Ref N8080127) and Superscript II (Invitrogen, Ref 18064-014).

qRT-PCR was performed on a Lightcycler 480 (Roche, Mannheim, Germany) using SYBR green I Master (Roche, Ref 04887352001). Data was analyzed using LinRegPCR software. Primer sequences are found in Table S2. We designed our primers to generate a single amplicon per PCR reaction. We verified the specificity of our qPCR primers by performing melting curve analysis, agarose electrophoresis separation and sequencing the amplicons. This confirmed that all qPCR products represented only one amplicon with the correct sequence.

### Live-cell analysis – confluence, cell death and apoptosis

10^5^ NRVMs were plated on 24-well plates coated with fibronectin. At 24 h after transfection, medium was replaced by medium containing 0.25 μg/ml recombinant Annexin V-ATTO-488 (Adipogen Life Sciences) and 300 nM YOYO3-612/631 (Invitrogen). Cell confluence was quantified over time by analyzing phase contrast images. Immediately after treatment, cells were incubated in an IncuCyte ZOOM System (Essen Bioscience, Ann Arbor, MI, USA) as previously described (Gelles and Chipuk, 2016). Experiments were conducted over 72 h with a scan every 24 h to avoid photobleaching of fluorescent reporters. Using the ×20 objective, 16 images per well were taken with data from phase contrast, green channel (Excitation: 440/480 nm; Emission: 504/544 nm) and red channel (Ex: 565/605 nm; Em: 625/705 nm) were collected. Fluorescent events were processed and analyzed using the IncuCyte ZOOM. Processing definitions for Annexin-V(488) and YOYO3(612/631) labeled cells were defined as follows: Channel: Green; Top-Hat; Radius: 100 μM, Threshold: 2.0 RCU; Edge Sensitivity: 0; Pixel Adjust: 0; Area: >9 μm; Eccentricity: undefined; Mean Intensity: undefined; Integrated Intensity: undefined. Channel: Red; Top-Hat; Radius: 100 μM, Threshold: 2.0 RCU; Edge Sensitivity: 0; Pixel Adjust: 0; Area: >11 μm; Eccentricity: undefined; Mean Intensity: undefined; Integrated Intensity: undefined. The total fluorescent areas of Annexin V and YOYO-3 in each image were analyzed to determine the apoptotic and dead cells relative to the whole image area. Those results were normalized to cell confluence in order to minimize the differences in cell density after plating.

### Immunostaining

10^5^ NRVMs were seeded over fibronectin-coated glass coverslips on 24-well plates. At 48 h after transfection, cells were fixed in 4% paraformaldehyde (PFA), permeabilized with PBS 0.1% Triton X-100, block in 4% goat serum and incubated overnight with the primary antibodies in a wet chamber at 4°C. Secondary antibodies were incubated for 1 h at room temperature, followed by 1 h of DAPI staining. Cell membranes were stained with Wheat Germ Agglutinin, Alexa Fluor™ 488 Conjugate (Invitrogen, Ref W11261) following manufacturer's protocol. Images were acquired with a Leica TCS SP8 X unit mounted on a Leica DMI6000 inverted microscope. Cell surface was quantified with the software Leica Application Suite X (LAS X). Antibodies and dyes references are found in Table S3.

### Protein isolation and western blotting

For protein isolation, cells were scraped and homogenized by sonication in RIPA buffer (50 mM Tris-HCl pH 8, 150 mM NaCl, 1% NP-40, 0.2% sodium deoxycholate, 0.1% SDS, 1 mM Na_3_VO_4_ and 1 mM PMSF) supplemented with protease inhibitor cocktail (Roche, Ref 11836170001). Protein concentration was measured with a BCA assay (Pierce, Ref 23227, Rockford, IL, USA). Western blotting was performed following standard protocols. Proteins were separated by SDS-PAGE, transferred to PVDF membranes (Bio-Rad, Ref 17001917, Hercules, CA, USA), block in TBST 4% milk and incubated with primary antibodies overnight at 4°C. Membranes were subsequently incubated with HRP-conjugated secondary antibodies for 1 h at room temperature. Western blots were developed with ECL prime western blotting detection agent (Amersham Biosciences, Ref RPN2232, Buckinghamshire, UK) in ImageQuant LAS 4000 (GE Healthcare Life Sciences). Western blots were quantified using Fiji (ImageJ). Antibody references are listed in Table S3.

### Electrophysiology data acquisition and analysis

Na^+^ current (*I*_Na_) and L-type Ca^2+^ current (*I*_CaL_) were measured with the ruptured patch-clamp technique, using an Axopatch 200B amplifier (Molecular Devices, San Jose, CA, USA). Voltage control, data acquisition and analysis of currents were performed with pClamp10.6/Clampfit (Molecular Devices, San Jose, CA, USA). Borosilicate glass patch pipettes (Harvard Apparatus, Holliston, MA, USA) with a tip resistance of 2–2.5 MΩ were used. Series resistance (Rs) and cell membrane capacitance (Cm) were compensated for 80%. Current densities were calculated by dividing current amplitude by Cm. Cm was determined by dividing the decay time constant of the capacitive transient in response to 5 mV hyperpolarizing steps from −40 mV, by the Rs. *I*_Na_ and *I*_CaL_ were filtered at 5 kHz and 2 kHz, respectively. *I*_Na_ was digitized at 40 kHz, while *I*_CaL_ was digitized at 20 kHz.

### Na^+^ current measurements

*I*_Na_ was measured in single cells using a pipette solution containing (in mM): 3.0 NaCl, 133 CsCl, 2.0 MgCl_2_, 2.0 Na_2_ATP, 2.0 TEACl, 10 EGTA, 5.0 HEPES; pH 7.3 (CsOH). Cells were superfused with a bath solution containing (in mM): 130 NaCl, 20 CsCl, 1.8 CaCl_2_, 1.2 MgCl_2_, 11.0 glucose, 5.0 HEPES, 0.005 nifedipine; pH 7.4 (CsOH). *I*_Na_ was measured at room temperature in response to depolarizing voltage steps from a holding potential of −100 mV (cycle length of 5 s). *I*_Na_ was defined as the difference between peak and steady state current (at 50 ms). Voltage dependence of activation and inactivation curves were fitted with a Boltzmann function (*y*=[1+exp{(*V−V*_1/2_)/*k*}]^−1^), where *V*_1/2_ is the half-maximal voltage of (in)activation and *k*, the slope factor*.* Potentials were not corrected for the estimated change in liquid junction potential.

### L-type Ca^2+^ current measurements

*I*_CaL_ was measured in single cells at 36°C. The pipette solution contained (in mM): 140 CsCl, 5 K_2_-ATP, 10 HEPES, 10 EGTA; pH 7.2 (CsOH). The external solution contained (in mM): 145 TEACl, 5.4 CsCl, 1.8 CaCl_2,_ 1 MgCl_2_, 5.5 glucose, 0.2 4,4′-diisothiocyano-2,2′-stilbenedisulfonic acid (DIDS), 5 HEPES; pH 7.4 (CsOH). *I*_CaL_ was measured in response to depolarizing voltage steps from a holding potential of −60 mV (cycle length of 5 s) and it was defined as the difference between peak and steady state current (at 300 ms). Voltage dependence of activation and inactivation curves were fitted with Boltzmann function (*y*=[1+exp{(*V*−*V*_1/2_)/*k*}]^−1^), where *V*_1/2_ is the half-maximal voltage of (in)activation and *k*, the slope factor*.* Potentials were corrected for the estimated change in liquid junction potential (11 mV).

### Statistical analysis

Data was analyzed using GraphdPad Prism 9 (GraphPad, San Diego, CA, USA). Data is presented as mean±s.d. unless otherwise stated. Results were analyzed with appropriate statistical tests, as indicated in the respective figure legends. A value of *P*<0.05 was considered statistically significant.

## Supplementary Material

Supplementary information

Reviewer comments
