# Supplementary material for: Inhibition of minor intron splicing reduces Na+ and Ca2+ channel expression and function in cardiomyocytes
Source: J Cell Sci. 2022 Jan 7;135(1):jcs259191. doi: 10.1242/jcs.259191 (PMC8767276; doi:10.1242/jcs.259191)
Supplement: Supplementary information [file joces-135-259191-s1.pdf]

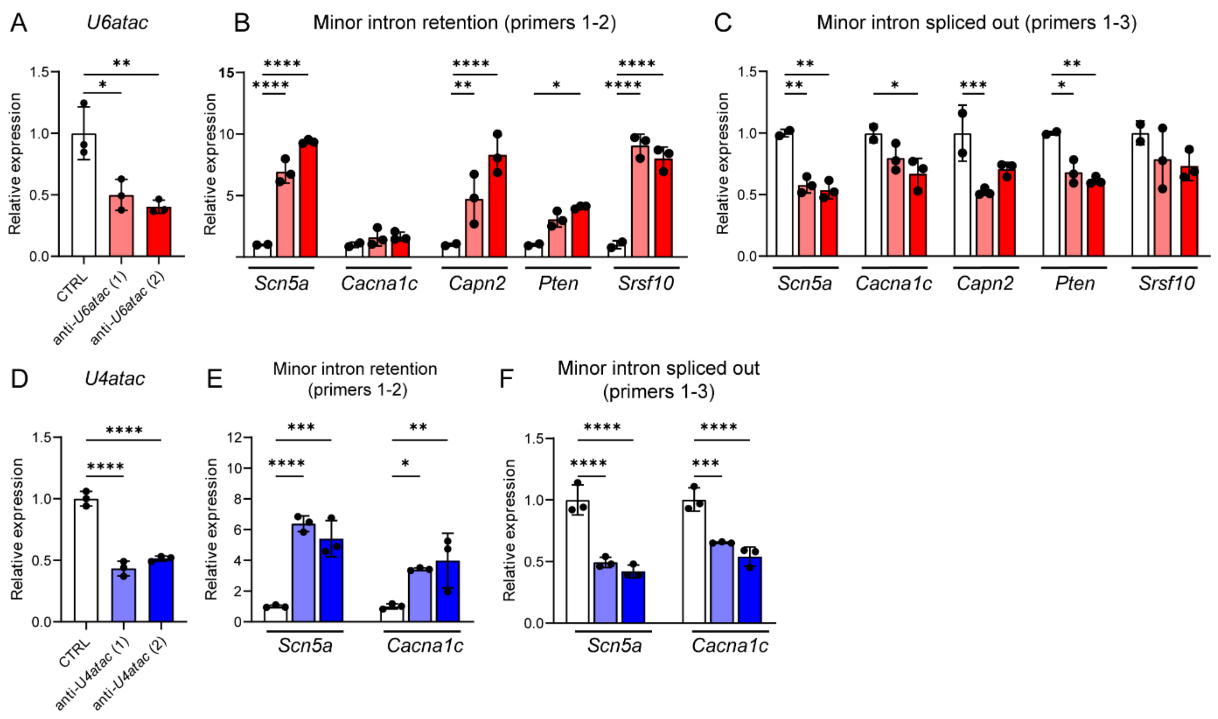

Fig. S1. Specificity of anti-*U6atac* and anti-*U4atac* gapmeRs. (A) Relative expression of the minor splicing component *U6atac*, (B) minor introns and (C) minor intron-flanking exons 24h after transfection with either a non-targeting control or 2 different anti-*U6atac* gapmeRs. (D) Relative expression of the minor splicing component *U4atac*, (E) minor introns and (F) minor intron-flanking exons 24h after transfection NRVMs with either a non-targeting control or 2 different anti-*U4atac* gapmeRs. Reference gene: *Hprt1*. Data are presented as mean ± s.d. Unpaired Student's t-test; * $p < 0.05$; ** $p < 0.01$; *** $p < 0.001$, **** $p < 0.0001$.

Journal of Cell Science • Supplementary information

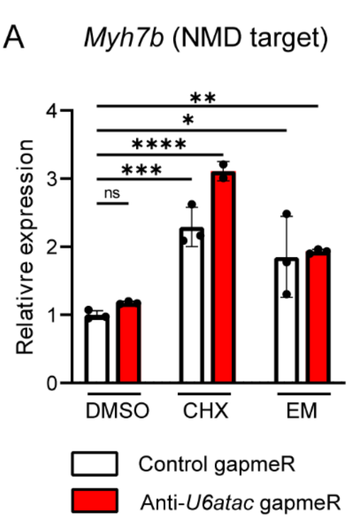

Fig. S2. NMD inhibition do not rescue the effect of minor intron retention. (A) Relative expression of the NMD-target *Myh7b*, (B) minor intron of *Scn5a and* (C) minor intron of *Cacna1c*. Cells were incubated for 3h with DMSO (control), 300 µg/µl cycloheximide (CHX) or 150 µg/µl emetine (EM) 24h after transfection with gapmeRs. Reference gene: *Hprt1*. Data are mean ± s.d. One-way ANOVA, followed by Holm-Sidak test for post hoc analyses; * $p < 0.05$; ** $p < 0.01$; *** $p < 0.001$, **** $p < 0.0001$.

Journal of Cell Science • Supplementary information

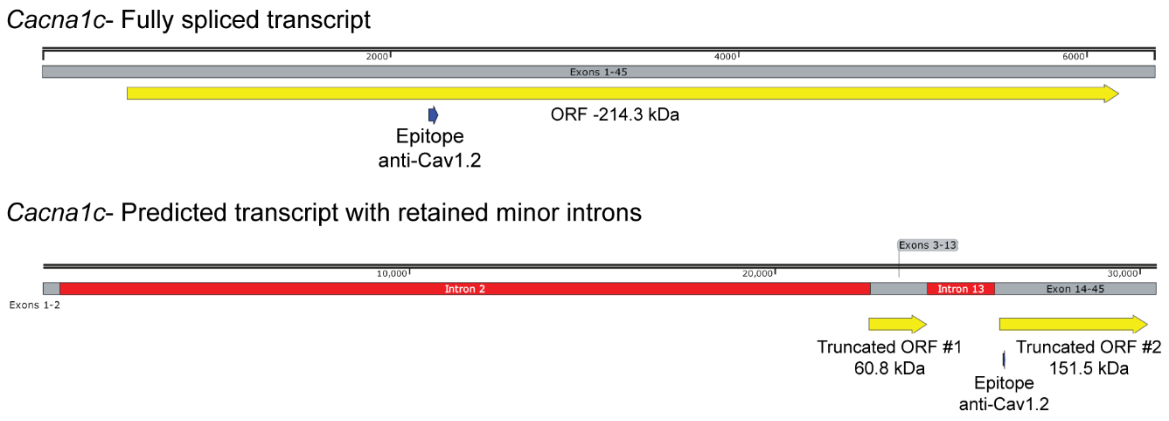

Fig. S3. Predicted size of the transcript and open reading frames of *Cacna1c* after minor intron retention.

Journal of Cell Science • Supplementary information

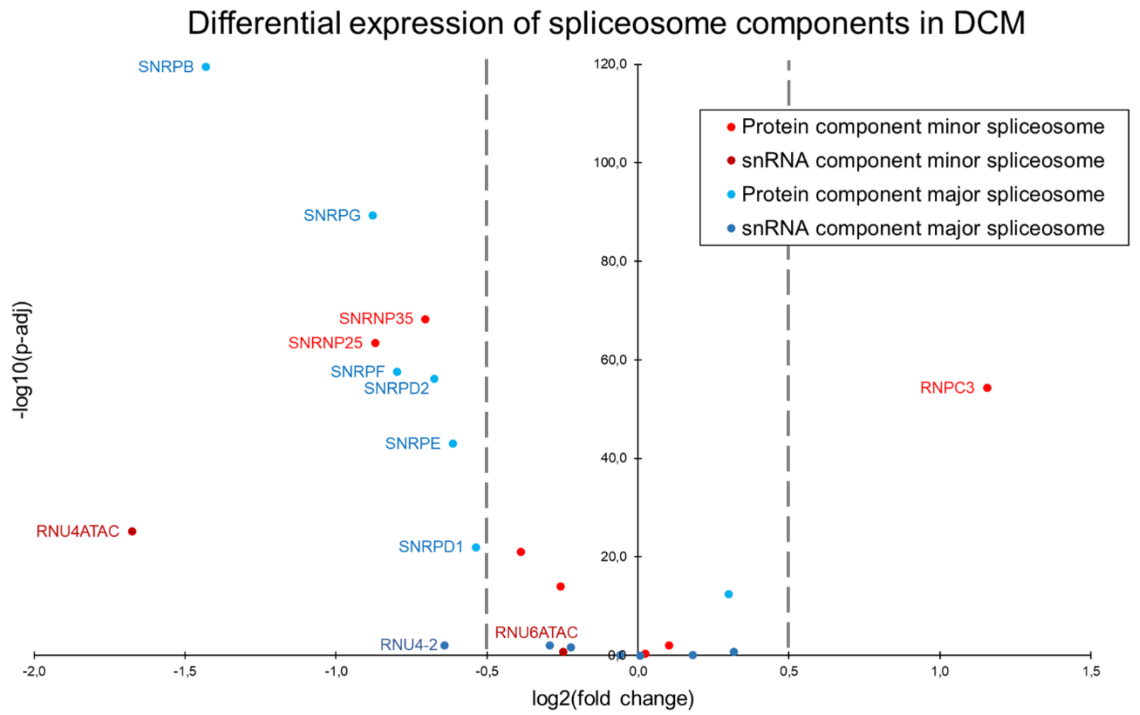

Fig. S4. Volcano plot showing differential expression of the protein and snRNA components of the minor and the major spliceosome in 100 non-diseased (CON) and 128 dilated cardiomyopathy (DCM) hearts. Only genes with TPM ≥ 0.1 were considered. Genes with absolute log2 fold change ≥ 0.58 (dashed lines) and adjusted p-value ≤ 0.05 were considered significantly differentially expressed.

Journal of Cell Science • Supplementary information

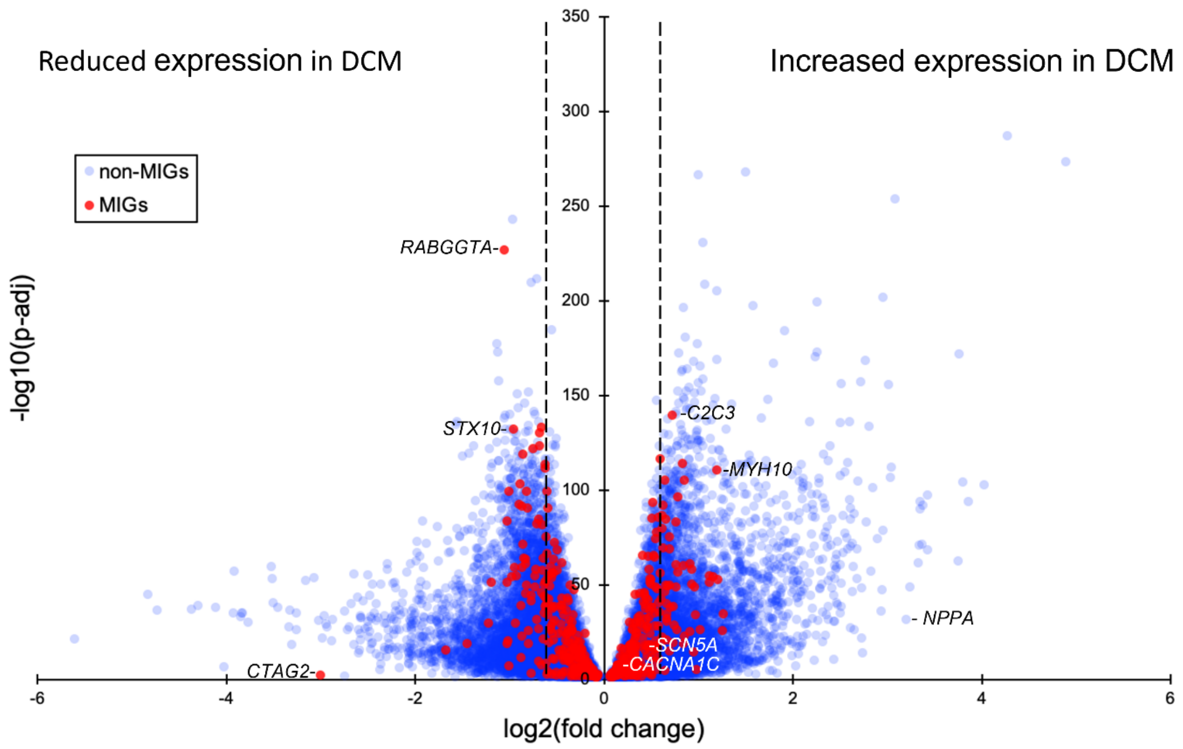

Fig. S5. Volcano plot showing differential expression of minor-intron containing genes (MIGs) and genes non-containing minor introns (non-MIGs) in 100 non-diseased (CON) and 128 dilated cardiomyopathy (DCM) hearts. Only genes with TPM ≥ 0.1 were considered. Genes with absolute log2 fold change ≥ 0.58 (dashed lines) and adjusted p-value ≤ 0.05 were considered significantly differentially expressed.

Table S1. Antisense LNA GapmeR sequences

GapmeR	Sequence 5’ -3’
Non-targeting negative control	AACACGTCTATACGC
Anti-*U6atac* (1)	CAATGCCTTAACCGTA
Anti-*U6atac* (2)	TGCCTTAACCGTATG
Anti-*U4atac* (1)	GTAGCGCAACCCCAAG
Anti-*U4atac* (2)	TAACCGATGCAGGTGT
FAM-labeled control	/56-FAM/AACACGTCTATACGC

Journal of Cell Science • Supplementary information

Table S2. Primer sequences

Primer	Sequence	Annealing on exon/intron (total number)
**EEF1E1 Fw**	TCCAGTAAAGAAGACACCCAGA	
**EEF1E1 Rv**	GACAAAACCAGCGAGACACA	
**GAPDH Fw**	GGTGGACCTCATGGCCTACA	
**GAPDH Rv**	CTCTCTTGCTCTCAGTATCCTTGCT	
**HPRT1 Fw**	TGACTATAATGAGCACTTCAGGGATTT	
**HPRT1 Rv**	CGCTGTCTTTTAGGCTTTGTACTTG	
**MYH7B Fw**	CTCAAGCGGGAGAACAAGAATC	
**MYH7B Rv**	CTGAGGCTGACCTGGTCTGTAA	
**U4ATAC Fw**	ATCCTTTTCTTGGGGTTGCG	
**U4ATAC Rv**	CGATGCAGGTGTGTTGTCAG	
**U6ATAC Fw**	GGTTAGCACTCCCCTTGACA	
**U6ATAC Rv**	AAGTAGGTGGCAATGCCTTAACC	
**U11 Fw**	GCGTGCGGAATCGACATCAAG	
**U11 Rv**	AAGGGCGCCGGGACCAACG	
**U12 Fw**	ATAACGATTCGGGGTGACGC	
**U12 Rv**	AGGCATCCCGCAAAGTAGGC	
**SCN5A 1 Fw**	TCTTCCGGTTCAGTGCCACC	Exon 3 (28)
**SCN5A 2 Rv**	CAGGACAGATGCGGATTAAGAGC	Intron 3 (27)
**SCN5A 3 Rv**	GGATGGTGCACATGATGAGCATG	Exon 4 (28)
**SCN5A 4 Rv**	AGGATGACGATGATGCTGTCG	Exon 15 (28)
**SCN5A 5 Fw**	ACCGACTCCTTCTCTCTTCTTC	Intron 14 (27)
**SCN5A 6 Fw**	GAGGAGATGCTGCAGGTCGG	Exon 14 (28)
**CACNA1C 1 Fw**	TGGGATCATGGCTTATGGCGGC	Exon 13 (45)
**CACNA1C 2 Rv**	GGCAGCATCAGCACCAAAGG	Intron 13 (44)
**CACNA1C 3 Rv**	ATCAGCCAGGTTGTCCACCG	Exon 14 (45)
**CACNA1C 4 Fw**	GAAGTTGTGTCCTCACCGTG	Exon 35 (45)
**CACNA1C 5 Rv**	TCACAAAGGCAAACAGGTAGC	Intron 35 (44)
**CACNA1C 6 Rv**	GGCAAACAGTGTAGCATTGAAC	Exon 36 (45)
**CAPN2 1 Fw**	GGCTTCAGCATCGAGACCTG	Exon 14 (21)
**CAPN2 2 Rv**	CAGGCTGCCTGTCCACA	Intron 14 (20)
**CAPN2 3 Rv**	CGTCCAGAGGATGTAGAACTCC	Exon 15 (21)
**CAPN2 4 Fw**	ATTGGAGATGGATTCAGAAGGC	Exon 16 (21)
**CAPN2 5 Rv**	GCTAAGGGTCTGAGCGAC	Intron 16 (20)
**CAPN2 6 Rv**	CTAGAACTCTTCTCAAGATGGTCTG	Exon 17 (21)
**PTEN 1 Fw**	TCAGCCACAGGCTCCCAGAC	Exon 1 (9)
**PTEN 2 Rv**	TTCGCATCCGTCTACTCCCACG	Intron 1 (8)
**PTEN 3 Rv**	ACACCTTCAAGTCTTTCTGCAGG	Exon 2 (9)
**PTEN 4 Fw**	GTGTGTGGTGACATCAAAGTAG	Exon 7 (9)
**PTEN 5 Rv**	ACTAATCTCCTAACCAAAGGCAC	Intron 7 (8)
**PTEN 6 Rv**	TCCTCTGGTCCTGGTATGAAG	Exon 8 (9)
**SRSF10 1 Rv**	GCGTCTTCAGCATCACGAACATC	Exon 3 (5)

**SRSF10 2 Fw**	GAGTTGTTTCAGACTTCACAAGCC	Intron 2 (4)
**SRSF10 3 Fw**	AGATTTACGTCGGGAATTTGGTCG	Exon 2 (5)
**SRSF10 4 Rv**	TACGCCGTGGTCTTCCAG	Exon 5 (5)
**SRSF10 5 Fw**	TGTGTATCTTGGATGCTTCATTAAGG	Intron 4 (4)
**SRSF10 6 Fw**	GGAGGAGATCAAGGAGTCGG	Exon 4 (5)

Journal of Cell Science • Supplementary information

Table S3. Antibodies and dyes

Antibody	Dilution	Reference
Anti-tubulin	1:5000 (WB)	GeneTex Cat No. GTX628802-01
Anti-Na_v1.5(SCN5A)	1:200 (WB)	Sigma S0819
Anti-Ca_v1.2 (CACNA1C)	1:200 (WB)	Alomone Cat No. ACC-003
Anti-rabbit-HRP	1:10000 (WB)	Amersham NA9340V
Anti-mouse-HRP	1:10000 (WB)	Amersham NA9310V
Anti-Vimentin	1:1000 (IF)	Abcam AB92537
Anti-alpha-actinin	1:750 (IF)	Sigma A781
Goat anti-rabbit-488	1:250 (IF)	Invitrogen A-11008
Goat anti-mouse-647	1:250 (IF)	Invitrogen A-21235
Wheat germ-agglutinin-488	1:200 (IF)	Invitrogen W11261
DAPI	1:1000 (IF)	Invitrogen D1306

Table S4. Sodium current properties in neonatal rat ventricular myocytes (NRVMs) 48h after transfection with control or anti-*U6atac* gapmeR.

	Control	n	Anti-*U6atac*	n
Current density				
**I_{Na} (pA/pF)**	-198.8±36.4	12	-82.3±18.5*	12
Activation				
**$V_{1/2}$ (mV)**	-43.7±1.6	12	-39.3±1.1*	12
**k (mV)**	6.5±0.3	12	7.5±0.2*	12
Inactivation				
**$V_{1/2}$ (mV)**	-90±2.7	10	-89.1±1.4	10
**k (mV)**	-5.9±0.3	10	-6.9±0.5	10

I_{Na}, sodium current density at -25 mV; $V_{1/2}$ of (in)activation, half-voltage of (in)activation; k, slope of the (in)activation curve; n, number of cells. * $p < 0.05$ *vs* control; unpaired Student's t-test or Mann-Whitney test when data were not normally distributed.

Table S5. L-type calcium current properties in neonatal rat ventricular myocytes (NRVMs) 48h after transfection with control or anti-*U6atac* gapmeR.

	Control	n	Anti-*U6atac*	n
Current density				
**I_{CaL} (pA/pF)**	-16.0±1.7	13	-10.3±1.0*	12
Activation				
**$V_{1/2}$ (mV)**	-13.0±0.8	13	-14.6±1.1	12
***k* (mV)**	7.3±0.2	13	7.3±0.3	12
Inactivation				
**$V_{1/2}$ (mV)**	-38.3±1.3	10	-37.7±0.7	9
***k* (mV)**	-6.0±0.3	10	-6.1±0.2	9

I_{CaL}, L-type calcium current density at 0 mV; $V_{1/2}$ of (in)activation, half-voltage of (in)activation; *k*, slope of the (in)activation curve; n, number of cells. * $p < 0.05$ *vs* control; unpaired Student's t-test.

Table S6.

Click here to download Table S6

Journal of Cell Science • Supplementary information
