# Supplementary material for: Inhibition of minor intron splicing reduces Na+ and Ca2+ channel expression and function in cardiomyocytes
Source: J Cell Sci. 2022 Jan 7;135(1):jcs259191. doi: 10.1242/jcs.259191 (PMC8767276; doi:10.1242/jcs.259191)
Supplement: Reviewer comments [file joces-135-259191_review_history.pdf]

Inhibition of minor intron splicing reduces Na^+ and Ca2+ channel expression and function in cardiomyocytes

Pablo Montañés-Agudo, Simona Casini, Simona Aufiero, Auriane C. Ernault, Ingeborg van der Made, Yigal M. Pinto, Carol Ann Remme and Esther E. Creemers

DOI: 10.1242/jcs.259191

Editor: Maria Carmo-Fonseca

Review timeline

Original submission:	27 July 2021
Editorial decision:	18 August 2021
First revision received:	28 October 2021
Accepted:	26 November 2021

Original submission

First decision letter

MS ID#: JOCES/2021/259191

MS TITLE: Minor intron splicing regulates Na+ and Ca2+ channel expression and function in cardiomyocytes

AUTHORS: Pablo Montañés-Agudo, Simona Casini, Simona Aufiero, Auriane C. Ernault, Ingeborg van der Made, Yigal M. Pinto, Carol Ann Remme, and Esther E. Creemers
ARTICLE TYPE: Research Article

We have now reached a decision on the above manuscript.

To see the reviewers' reports and a copy of this decision letter, please go to: https://submit-jcs.biologists.org and click on the 'Manuscripts with Decisions' queue in the Author Area. (Corresponding author only has access to reviews.)

As you will see, the reviewers raise a number of substantial criticisms that prevent me from accepting the paper at this stage. They suggest, however, that a revised version might prove acceptable, if you can address their concerns. If you think that you can deal satisfactorily with the criticisms on revision, I would be pleased to see a revised manuscript. We would then return it to the reviewers.

We are aware that you may be experiencing disruption to the normal running of your lab that makes experimental revisions challenging. If it would be helpful, we encourage you to contact us to discuss your revision in greater detail. Please send us a point-by-point response indicating where you are able to address concerns raised (either experimentally or by changes to the text) and where you will not be able to do so within the normal timeframe of a revision. We will then provide further guidance. Please also note that we are happy to extend revision timeframes as necessary.

Please ensure that you clearly highlight all changes made in the revised manuscript. Please avoid using 'Tracked changes' in Word files as these are lost in PDF conversion.

© 2021. Published by The Company of Biologists under the terms of the Creative Commons Attribution License (https://creativecommons.org/licenses/by/4.0/).

I should be grateful if you would also provide a point-by-point response detailing how you have dealt with the points raised by the reviewers in the 'Response to Reviewers' box. Please attend to all of the reviewers' comments. If you do not agree with any of their criticisms or suggestions please explain clearly why this is so.

Reviewer 1

Advance summary and potential significance to field

JOCES-2021-25919

Minor intron splicing regulates Na+ and Ca2+ channel expression and function in cardiomyocytes

Pablo Montañés-Agudo, Simona Casini, Simona Aufiero, Auriane C. Ernault, Ingeborg van der Made, Yigal M. Pinto, Carol Ann Remme, Esther E. Creemers

In order to investigate whether the minor spliceosome regulates electrophysiological properties of cardiomyocytes by controlling the expression of minor-intron containing genes (MIGs) or not, the authors tested and found that a large proportion (582/699) of MIGs is expressed in the human heart. Pathway analysis revealed an enrichment for genes of electrophysiological processes, including the two crucial cardiac genes:
SCN5A and CACNA1C. They knocked down the essential minor spliceosome component U6atac in neonatal rat ventricular myocytes and found that loss of U6atac led to robust minor intron retention within SCN5A and CACNA1C. This results in reduced protein levels of Nav1.5 and Cav1.2. Lastly the authors demonstrated that the expression of minor spliceosome components in dilated cardiomyopathy human hearts.

Comments for the author

Minor intron splicing regulates Na+ and Ca2+ channel expression and function in cardiomyocytes
Pablo Montañés-Agudo, Simona Casini, Simona Aufiero, Auriane C. Ernault, Ingeborg van der Made, Yigal M. Pinto, Carol Ann Remme, Esther E. Creemers

In order to investigate whether the minor spliceosome regulates electrophysiological properties of cardiomyocytes by controlling the expression of minor-intron containing genes (MIGs) or not, the authors tested and found that a large proportion (582/699) of MIGs is expressed in the human heart. Pathway analysis revealed an enrichment for genes of electrophysiological processes, including the two crucial cardiac genes:
SCN5A and CACNA1C. They knocked down the essential minor spliceosome component U6atac in neonatal rat ventricular myocytes and found that loss of U6atac led to robust minor intron retention within SCN5A and CACNA1C. This results in reduced protein levels of Nav1.5 and Cav1.2. Lastly the authors demonstrated that the expression of minor spliceosome components in dilated cardiomyopathy human hearts.
The findings are potentially interesting. However, intron retention caused by U6atac, a known essential factor for minor intron, is easily predictable and it does not have novel findings. Although the finding that the expression of minor spliceosome components in dilated cardiomyopathy human hearts is interesting, the authors did not go further to obtain mechanistic insights. Results from other experiments are necessary to strengthen the conclusion.

Specific comments:
1) With CACNA1C, the authors mention about a possibility for NMD. The authors should determine and mention whether NMD takes place by intron retention of all the gene products they checked.
2) In all Figures with qRT-PCR, it would be better to show agarose or polyacrylamide gel electrophoresis patterns in order to demonstrate they amplified the target products specifically.
3) In Figure 4, the authors mention a smaller product can be detected. Does this product meet with the size of the protein that are produced from minor-intron retained mRNA? Even this is the case, it is required to test whether the product from intron containing RNA is detected by this antibody or not. If the authors could demonstrate their hypothesis is correct, they should then determine

© 2021. Published by The Company of Biologists under the terms of the Creative Commons Attribution License (https://creativecommons.org/licenses/by/4.0/).

whether the smaller product work as a dominant-negative protein that causes reduction of L-type calcium current density or not.
4) In Figure 2C, the authors do not show smaller region of the gel. Did they detect the smaller product that they could see in Figure 4?
5) In is interesting that the authors demonstrate some of minor-spliceosomal factors are less expressed in dilated cardiomyopathy hearts. Is the expression of major spliceosomal components affected? Do the authors have any idea for this phenomenon to discuss?
6) In the title, the authors describe 'Minor intron splicing regulates'. Are those minor introns in SCN5A and CACNA1C pre-mRNAs alternatively spliced ones? If not, I think the title is not suitable, because those introns are constitutive introns that have to be removed anyway.

Reviewer 2

Advance summary and potential significance to field

While the paper does not establish a clear cause-effect relationship between minor spliceosome and physiologically relevant effects on heart electrophysiology, it raises and strongly supports the possibility. I can't think of something that is reasonable to ask the authors to do to make the paper closer to establishing cause and effect. In my opinion, what is presented is important since a role for the minor spliceosome is rarely considered and the case they make is new and convincing.

Comments for the author

The goal of this study is to determine the potential role of U12 (minor) introns and components of the U12 spliceosome in heart physiology. The justification is that a number of genes directly relevant to heart electrophysiology contain U12 introns. The authors used published RNA-seq data to determine that a high fraction of the U12 intron-containing genes (582 out of 699) are expressed in heart tissues. U12 intron genes were expressed at a moderately higher level than non U12 intron genes and GO analysis included enrichment for sodium and calcium channels. Anti U6atac gapmeRs were used to knock down (KD) U6atac snRNA up to 60% in culture neonatal rat ventricular cardiomyocytes. Five minor introns showed increased inclusion while major introns had no retention. The cells were relatively unaffected with regard to viability. SCN5A protein levels were reduced ~50% and I50 was similarly reduced 59%. Levels of CACNA1C protein were also reduced and patch clamp analysis showed decreased ICaL density. Finally the authors use RNA-seq data from normal and failing hearts to show reductions as well as elevations of U12 spliceosome components. The results bring important attention to the potential role of minor intron splicing in regulating expression of genes particularly important to heart electrophysiology. While KD of an essential component of the U12 splicing machinery is an extreme physiological situation, the authors make a decent argument for modulation of U6atac levels as a physiologically regulated component. U6atac was a good choice for KD because it is a rate limiting component the levels of which are regulated. One issue with the manuscript is the lack of strong physiological relevance to human heart disease or heart disease models. Overall the message of the paper is important and the work is well done.

Reviewer 3

Advance summary and potential significance to field

In this report, authors investigated the expression and functional consequence of dysregulation of minor intron containing genes in cardiomyocytes. Functional enrichment of MIGs were analyzed based on cellular processes and pathways. Minor intron splicing was inhibited by targeted gene silencing of U6atac in neonatal rat ventricular myocytes, followed by targeted characterization of minor intron retention, protein expression and cellular sodium and calcium densities. In addition, the expression of minor intron splicing complex genes was evaluated in a dataset of heart failure vs. normal human heart. From these data, authors concluded that minor-intron splicing plays a role in cardiomyocyte physiology particularly electrophysiology and dysfunction in cardiomyopathies. The characterization of MIGs in terms of their function and regulation in the context of cardiac physiology is interesting, new and potentially important.

© 2021. Published by The Company of Biologists under the terms of the Creative Commons Attribution License (https://creativecommons.org/licenses/by/4.0/).

This is the first report of systemic characterization of cardiac MIGs and the overall results should be valuable to the field.

Comments for the author

However, the study appears to be somewhat incomplete in its current state.
1). The functional significance of MIGs in cardiac physiology and diseases were studied by evaluating minor spliceosome complex genes in a transcriptome dataset with a large cohort of DCM vs. control. It is interesting to note that U4atac was significantly downregulated in DCM heart relative to controls. However authors selected U6atac knockdown in NRVM to evaluate the functional impact. It would be very informative to demonstrate U6atac and U4atac inactivation has similar outcome in minor intron retention of the target genes. Without detailed information about the clinic features of DCM cohort, it is not possible to evaluate the correlation of these genes with clinic outcome in terms of cardiac sudden death or signs of arrhythmia, or other co-morbidities. In addition, the expression of MIGs and minor spliceosome genes should also be evaluated in available transcriptome datasets from rodent heart failure models where pathological features are usually more homogeneous and better controlled. In short, there are opportunities for authors to interrogate the dataset more rigorously to support the functional relevance of MIGs regulation via minor intron splicing in heart failure.

2). Authors performed validation studies in NRVM using targeted gapmeR mediated gene silencing. This is a very strong set of data indicating the impact of minor intron splicing on targeted gene expression and cellular function. In general, it is a requirement that more than one gapmeR should be tested to support the specificity, and the expression of other minor splicing complex genes should be evaluated. The limitation of NRVM due to their immature EP function should be discussed and acknowledged by the authors. While the utilization of adult myocytes would be more relevant, it is understandable that adult myocytes are not easily amendable to genetic manipulation in culture.
However, authors should evaluate the expression of minor intron splicing events in MIGs as well as the minor spliceosome specific complex genes in neonatal vs. adult hearts. EP maturation is an important part of cardiomyocyte maturation and reversal of this process is often viewed as part of the “fetal-like reprogramming” in diseased hearts. Therefore, having the expression data analyzed across cardiac maturation would add more significance to this finding.

3). It is interesting to note that PTEN is also a MIG and subjected to minor-intron retention upon minor-intron specific spliceosome inactivation. Since PTEN is a key molecule in PI3K/AKT signaling during cardiac hypertrophy, it would be very interesting to evaluate the impact on cardiomyocyte size and growth under hypertrophic stimulations. Authors measured confluency, morphology and apoptotic activity under normal growth condition. However, cell surface area can be measured under basal (serum starved) vs. post-hypertrophic stimulation (using PE or serum) to evaluate PTEN mediated hypertrophic regulation.

First revision

Author response to reviewers' comments

Response to Reviewer 1
In order to investigate whether the minor spliceosome regulates electrophysiological properties of cardiomyocytes by controlling the expression of minor-intron containing genes (MIGs) or not, the authors tested and found that a large proportion (582/699) of MIGs is expressed in the human heart. Pathway analysis revealed an enrichment for genes of electrophysiological processes, including the two crucial cardiac genes: SCN5A and CACNA1C. They knocked down the essential minor spliceosome component U6atac in neonatal rat ventricular myocytes and found that loss of U6atac led to robust minor intron retention within SCN5A and CACNA1C. This results in reduced protein levels of Nav1.5 and Cav1.2. Lastly the authors demonstrated that the expression of minor spliceosome components in dilated cardiomyopathy human hearts. The findings are potentially interesting. However, intron retention caused by U6atac, a known

© 2021. Published by The Company of Biologists under the terms of the Creative Commons Attribution License (https://creativecommons.org/licenses/by/4.0/).

essential factor for minor intron, is easily predictable and it does not have novel findings. Although the finding that the expression of minor spliceosome components in dilated cardiomyopathy human hearts is interesting, the authors did not go further to obtain mechanistic insights. Results from other experiments are necessary to strengthen the conclusion.
We thank the reviewer for her/his comments on the manuscript. We agree that the essential role of U6atac in minor splicing was previously characterized, however the novelty of our work relates to the fact that minor introns are not randomly distributed among genes, but are enriched in specific gene families such as voltage-gated sodium and calcium channels. As these channels are imperative for cardiac (electro)physiology we set out experiments to see whether changes in minor spliceosome components could affect sodium and calcium currents in the heart. This has, to the best of our knowledge, never been explored.

1) **With CACNA1C, the authors mention about a possibility for NMD. The authors should determine and mention whether NMD takes place by intron retention of all the gene products they checked.**

This is a good point. Indeed, NMD may eliminate transcripts in which the minor intron is retained. As suggested by the reviewer, we have now performed additional experiments in which we knocked- down U6atac in neonatal cardiomyocytes and at the same time inhibit the NMD pathway by adding the translation inhibitors cycloheximide (CHX) or emetine (EM). See figure below, which has been added to the manuscript as Figure S2 and on manuscript lines 156-163 and 437-439.

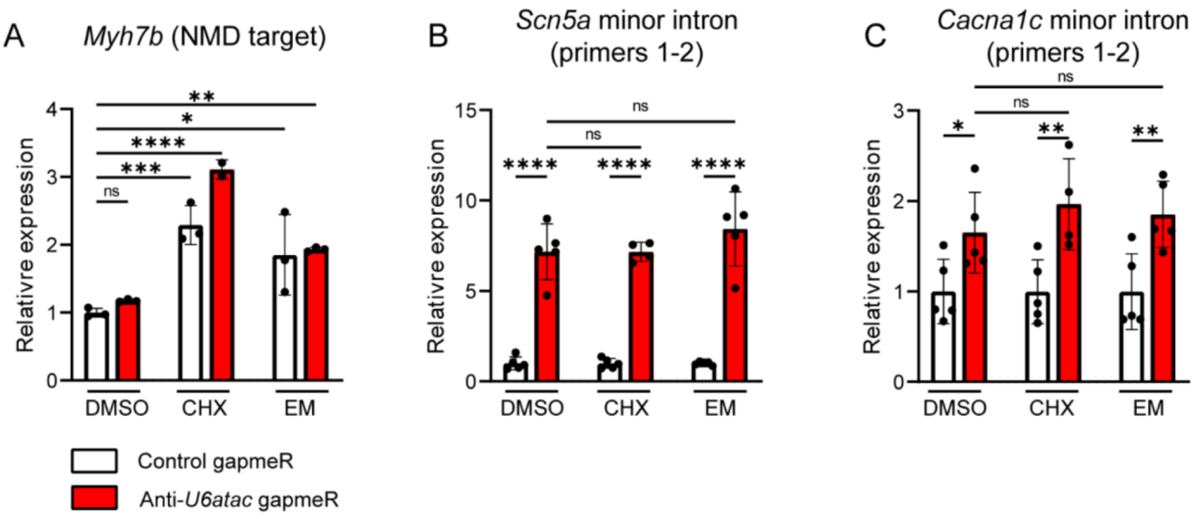

This shows that NMD inhibition does not further increase minor intron retention in *Scn5a* or *Cacna1c* (panel B and C) and thus mRNA levels of both genes, indicating that transcripts with minor intron retention are not targeted by NMD. We included *Myh7b* – a bone fide target of the NMD pathway in cardiomyocytes – as a positive control for the NMD inactivation (panel A). Since *Scn5a* and *Cacna1c* are the focus of our manuscript we limited our analysis to these two genes.

2) **In all Figures with qRT-PCR, it would be better to show agarose or polyacrylamide gel electrophoresis patterns in order to demonstrate they amplified the target products specifically**

We designed our primers to generate a single amplicon per PCR reaction. We verified the specificity of our qPCR primers by performing melting curve analysis, agarose electrophoresis separation and sequencing the amplicons. This confirmed that all qPCR products represented only one amplicon with the correct sequence. We added this claim in manuscript lines 452-455.

3) **In Figure 4, the authors mention a smaller product can be detected. Does this product meet with the size of the protein that are produced from minor-intron retained mRNA? Even this is the case, it is required to test whether the product from intron containing RNA is detected by this antibody or not. If the authors could demonstrate their hypothesis is**

© 2021. Published by The Company of Biologists under the terms of the Creative Commons Attribution License (https://creativecommons.org/licenses/by/4.0/).

correct, they should then determine whether the smaller product work as a dominant-negative protein that causes reduction of L-type calcium current density or not.

The reviewer raises an interesting point. The smaller product can not be explained by a truncated ORF. Two ORFs that theoretically can arise after retention of intron 2 are shown below, and they are respectively 60 kDa and 151 kDa in size. Only the 151 kDa protein contains the epitope for the antibody we used. The smaller product that we found in Figure 4 is approximately 100 kDa, and therefore does not meet the size of predicted protein produced from intron containing transcript. It is not clear whether the 100 kDa band arose from an aberrantly spliced isoform that was induced after minor splicing inhibition, or whether it represents something else. This point was added in Fig S3 and in the manuscript lines 224-228.

Follow-up studies in which the sequence and function of the protein are investigated would be interesting, but are to our opinion, outside the scope of this manuscript.

Cacna1c- Fully spliced transcript

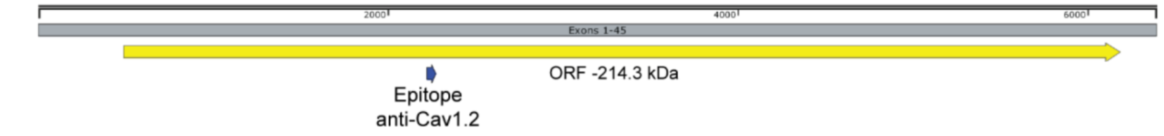

Cacna1c- Predicted transcript with retained minor introns

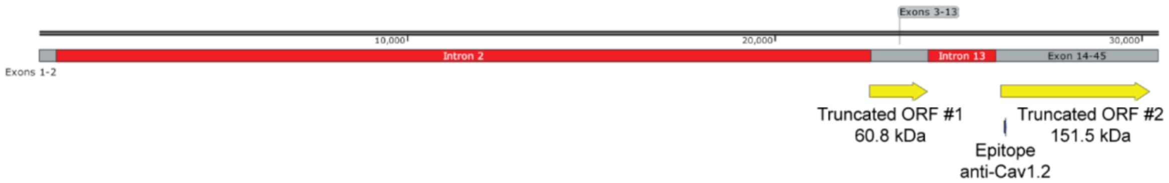

4) **In Figure 2C, the authors do not show smaller region of the gel. Did they detect the smaller product that they could see in Figure 4?**

We assume the Reviewer is referring to Fig 3C (new Fig 4C) instead of Fig 2C. We did not observe a smaller band in the Scn5a Western blots after U6atac knockdown, as shown in the gel below.

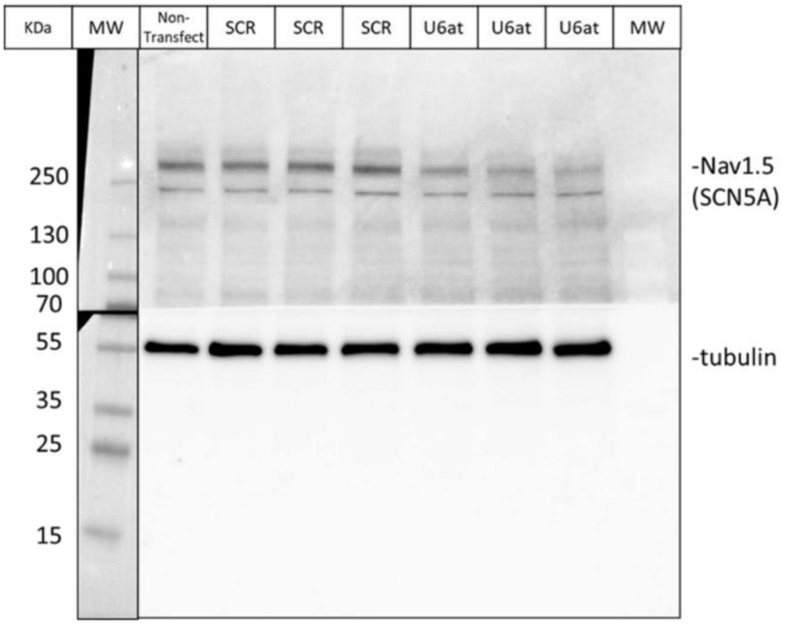

5) **It is interesting that the authors demonstrate some of minor-spliceosomal factors are less expressed in dilated cardiomyopathy hearts. Is the expression of major spliceosomal components affected? Do the authors have any idea for this phenomenon to discuss?**

© 2021. Published by The Company of Biologists under the terms of the Creative Commons Attribution License (https://creativecommons.org/licenses/by/4.0/).

We checked the differential expression of major spliceosome components in our dataset and plotted the data in Fig S4. As can be seen from this volcano plot (see below), some of the major spliceosome components are dysregulated as well in the human DCM hearts. This is in line with decreased splicing efficiency observed in heart failure (Kong et al., 2010), but also indicates that the dysregulations of minor spliceosome components is not a unique feature in DCM. We added these finding to the results section of the manuscript in lines 250-255.

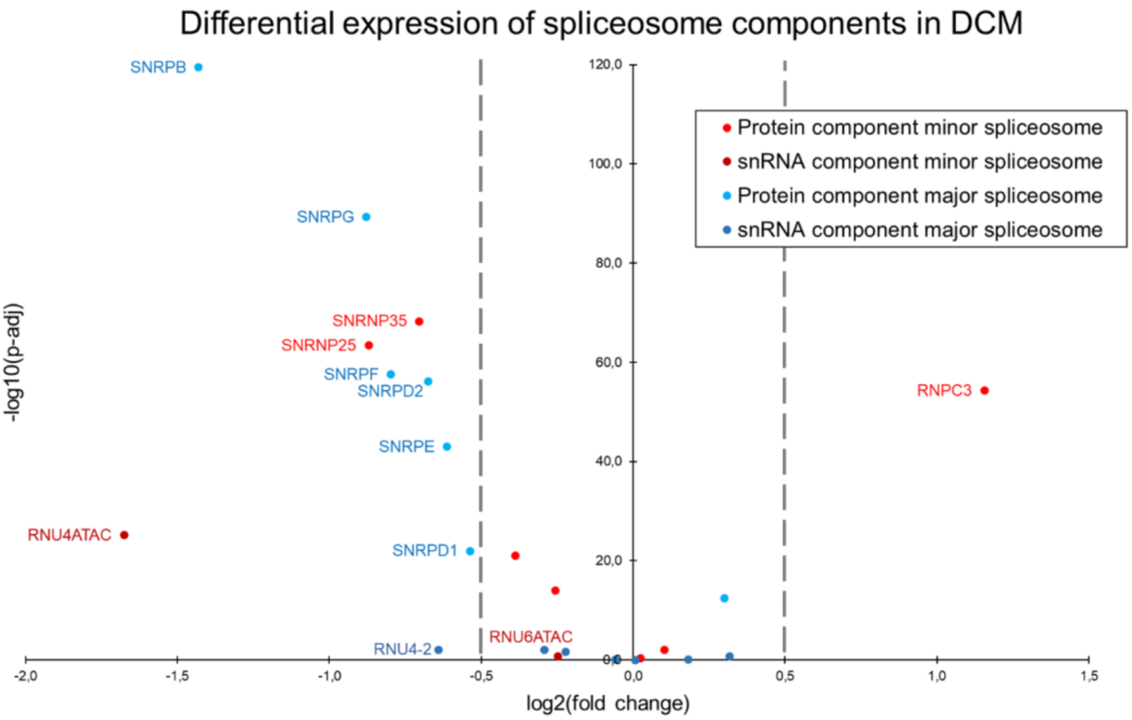

With respect to the second question raised, we are not aware of a study investigating the regulation of minor spliceosomeel components in the heart. However, for U6atac, it has been shown in Hela cells that the levels of this snRNA are increased due to the cell stress-activated kinase p38MAPK (Younis et al., 2013). Since p38 MAPK activation occurs in hypertrophic and hypoxia responses in cardiomyocytes, this may result in increased U6atac levels under these conditions. We mention the Younis et al. paper in the introduction section (lines 73-79), but do not have an explanation for the dysregulation of the core components of the major and the minor spliceosomes.

6) **In the title, the authors describe 'Minor intron splicing regulates'. Are those minor introns in *SCN5A* and *CACNA1C* pre-mRNAs alternatively spliced ones? If not, I think the title is not suitable, because those introns are constitutive introns that have to be removed anyway.**

We concur with the point that our title does not adequately reflect our findings. We therefore changed the word 'regulates' in our title. The new title therefore is: "Inhibition of minor intron splicing reduces Na^+ and Ca^{2+} channel expression and function in cardiomyocytes"

Response to Reviewer 2
Advance Summary and Potential Significance to Field:

While the paper does not establish a clear cause-effect relationship between minor spliceosome and physiologically relevant effects on heart electrophysiology, it raises and strongly supports the possibility. I can't think of something that is reasonable to ask the authors to do to make the paper closer to establishing cause and effect. In my opinion, what is presented is important since a role for the minor spliceosome is rarely considered and the case they make is new and convincing.
Comments for the Author:

© 2021. Published by The Company of Biologists under the terms of the Creative Commons Attribution License (https://creativecommons.org/licenses/by/4.0/).

The goal of this study is to determine the potential role of U12 (minor) introns and components of the U12 spliceosome in heart physiology. The justification is that a number of genes directly relevant to heart electrophysiology contain U12 introns. The authors used published RNA-seq data to determine that a high fraction of the U12 intron-containing genes (582 out of 699) are expressed in heart tissues. U12 intron genes were expressed at a moderately higher level than non U12 intron genes and GO analysis included enrichment for sodium and calcium channels. Anti U6atac gapmeRs were used to knock down (KD) U6atac snRNA up to 60% in culture neonatal rat ventricular cardiomyocytes. Five minor introns showed increased inclusion while major introns had no retention. The cells were relatively unaffected with regard to viability. SCN5A protein levels were reduced ~50% and I50 was similarly reduced 59%. Levels of CACNA1C protein were also reduced and patch clamp analysis showed decreased ICaL density. Finally the authors use RNA-seq data from normal and failing hearts to show reductions as well as elevations of U12 spliceosome components. The results bring important attention to the potential role of minor intron splicing in regulating expression of genes particularly important to heart electrophysiology. While KD of an essential component of the U12 splicing machinery is an extreme physiological situation, the authors make a decent argument for modulation of U6atac levels as a physiologically regulated component. U6atac was a good choice for KD because it is a rate limiting component the levels of which are regulated. One issue with the manuscript is the lack of strong physiological relevance to human heart disease or heart disease models. Overall the message of the paper is important and the work is well done.

We thank reviewer 2 for the favorable comments to the manuscript. In fact, we couldn't have worded the significance and limitations of our work better!

Response to Reviewer 3

In this report, authors investigated the expression and functional consequence of dysregulation of minor intron containing genes in cardiomyocytes. Functional enrichment of MIGs were analyzed based on cellular processes and pathways. Minor intron splicing was inhibited by targeted gene silencing of U6atac in neonatal rat ventricular myocytes, followed by targeted characterization of minor intron retention, protein expression and cellular sodium and calcium densities. In addition, the expression of minor intron splicing complex genes was evaluated in a dataset of heart failure vs. normal human heart. From these data, authors concluded that minor-intron splicing plays a role in cardiomyocyte physiology, particularly electrophysiology and dysfunction in cardiomyopathies. The characterization of MIGs in terms of their function and regulation in the context of cardiac physiology is interesting, new and potentially important. This is the first report of systemic characterization of cardiac MIGs and the overall results should be valuable to the field.

We appreciate the favorable and constructive comments of this reviewer.

1) **The functional significance of MIGs in cardiac physiology and diseases were studied by evaluating minor spliceosome complex genes in a transcriptome dataset with a large cohort of DCM vs. control. It is interesting to note that U4atac was significantly downregulated in DCM heart relative to controls. However, authors selected U6atac knockdown in NRVM to evaluate the functional impact. It would be very informative to demonstrate U6atac and U4atac inactivation has similar outcome in minor intron retention of the target genes.**

We initiated our experiments by knocking-down U6atac before we had the results of the DCM study. We chose U6atac because it has been described to be the limiting factor in minor splicing (Younis et al., 2013), but agree that it would be informative to measure the effect of U4atac knockdown as well. Therefore, we performed additional experiments in which we knocked-down U4atac by the use of 2 different gapmeRs and show that this induced minor intron retention in *Scn5a* and *Cacna1c*, equivalent to what occurs with U6atac knock-down (see figure below). We added the results in Fig S1A and in the manuscript lines 151-155.

© 2021. Published by The Company of Biologists under the terms of the Creative Commons Attribution License (https://creativecommons.org/licenses/by/4.0/).

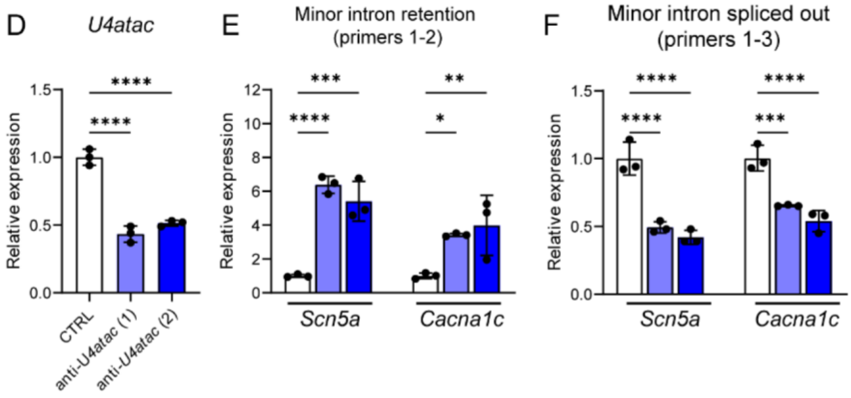

2) **Without detailed information about the clinic features of DCM cohort, it is not possible to evaluate the correlation of these genes with clinic outcome in terms of cardiac sudden death or signs of arrhythmia, or other co-morbidities. In addition, the expression of MIGs and minor spliceosome genes should also be evaluated in available transcriptome datasets from rodent heart failure models where pathological features are usually more homogeneous and better controlled. In short, there are opportunities for authors to interrogate the dataset more rigorously to support the functional relevance of MIGs regulation via minor intron splicing in heart failure.**

Unfortunately, we do not have detailed information about the clinical features of this DCM cohort, and therefore we are not evaluating correlations of the expression of these genes with signs of arrhythmias or sudden death.

With respect to the second point raised, we looked into another available RNA-seq dataset present in our lab, which interrogates the expression in the hearts of 4 control mice and 4 mice, 3 weeks after transverse aorta constriction (TAC) surgeries. In the figure below, we plotted the expression of minor spliceosome components in this cardiac hypertrophy model but did not find strong evidence for dysregulation (only *Pdcd7* was significantly upregulated). It would be interesting to investigate the expression of minor spliceosome components in a more severe model of heart failure in rodents, but we do not have this to our disposal. To keep the study focused, we did not include the 3-week TAC data to the manuscript.

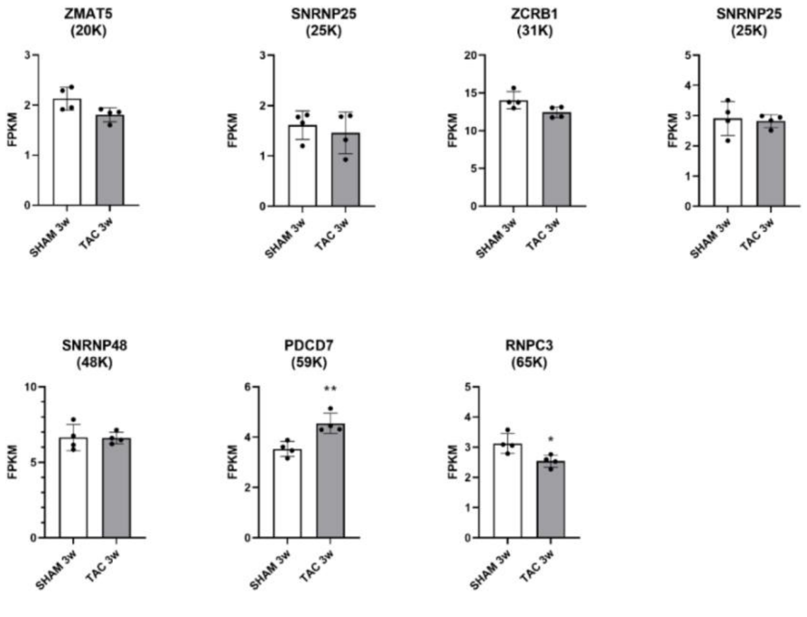

Expression of minor spliceosome components in a mouse model of cardiac hypertrophy, 3 weeks after transverse aortic constriction.

© 2021. Published by The Company of Biologists under the terms of the Creative Commons Attribution License (https://creativecommons.org/licenses/by/4.0/).

3) Authors performed validation studies in NRVM using targeted gapmeR mediated gene silencing. This is a very strong set of data indicating the impact of minor intron splicing on targeted gene expression and cellular function. In general, it is a requirement that more than one gapmeR should be tested to support the specificity, and the expression of other minor splicing complex genes should be evaluated.

Thank you for raising this important point. We have now performed additional experiments with two independent gapmeRs against U6atac (anti-U6atac (1) and anti-U6atac (2)). As shown below, both gapmeRs induced comparable levels of intron retention, further supporting the specificity of our findings. Results were added in Fig S1B and described in the manuscript, lines 151-155.

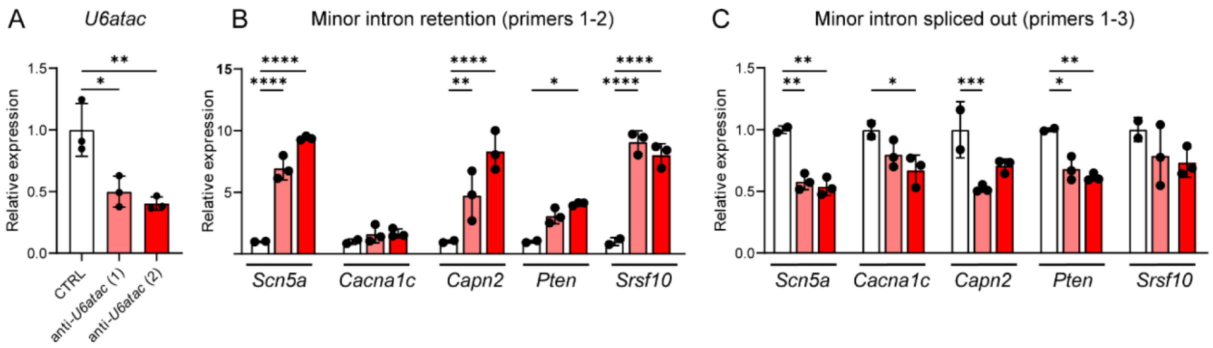

Of note, the gapmeRs were specific, since anti-*U6atac* only downregulated *U6atac* and anti-*U4atac* only *U4atac*, see figure below. We also measured the expression of *U11* and *U12*, two other snRNAs of the minor spliceosome, and we found a significant upregulation of *U11* when knocking down *U6atac* (red bars in figure below), but not *U4atac* (blue bars). It is unclear at this point whether the *U11* increase is a compensatory response to the loss of U6atac or whether it represents a secondary effect.

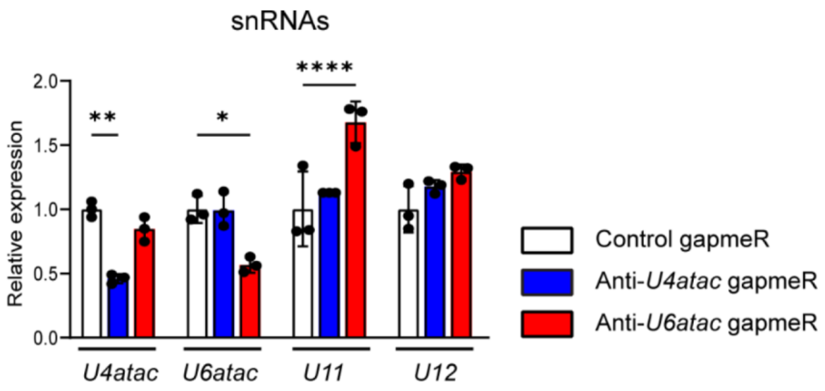

Expression of the snRNAs components of the minor spliceosome 24h after transfection of NRVMs with control, anti-4atac o anti-U6atac.

4) The limitation of NRVM due to their immature EP function should be discussed and acknowledged by the authors. While the utilization of adult myocytes would be more relevant, it is understandable that adult myocytes are not easily amendable to genetic manipulation in culture.

We acknowledge the limitations of NRVMs in terms of maturity, but as indicated by the reviewer adult myocytes are not amendable to transfections and prolonged culturing, so we did not have a good alternative. Particularly the expression of alternative splice isoforms of the voltage gated channels in adult versus neonatal hearts may lead to differences in EP function. However, as described in more detail below, the minor intron is present in fetal and adult splice isoforms of *SCN5A* and *CACNA1C*.

© 2021. Published by The Company of Biologists under the terms of the Creative Commons Attribution License (https://creativecommons.org/licenses/by/4.0/).

During maturation, *SCN5A* switches from the neonatal isoform containing exon 6A to the adult isoform containing exon 6B. This alternative splice event creates a 7 amino acids difference that alters the kinetic properties of the sodium channel (Onkal et al., 2008). The minor intron of *SCN5A* is intron 3, and is thus located upstream of the alternative spliced exons 6A and 6B. The L-type calcium current also differs between immature and mature cardiomyocytes due to alternative splicing of *CACNA1C*. In neonatal hearts, there are more transcripts including exon 21, which creates an isoform not able to conduct any Ca^{2+} (Hu et al., 2016). Exons 31 and 32 are mutually exclusive and differ in adult versus fetal isoforms (Diebold et al., 1992). Exon 33 presents an alternative splice site that generates a truncated and dominant negative isoform called CaV1.233L, which is more abundant in neonatal than in adult cardiomyocytes (Liao et al., 2015). All these alternative splice events occur downstream of the minor introns of *CACNA1C* (which represent introns 2 and 13).

Hence, since the minor introns are present in fetal and mature isoforms of SCN5*A* and *CACNA1C*, inhibition of minor splicing is expected to similarly affect sodium and L-type calcium currents in fully mature cardiomyocytes. We added a brief paragraph to acknowledge this in the discussion section, lines 333-342.

5) **Authors should evaluate the expression of minor intron splicing events in MIGs as well as the minor spliceosome specific complex genes in neonatal vs. adult hearts. EP maturation is an important part of cardiomyocyte maturation and reversal of this process is often viewed as part of the "fetal-like reprogramming" in diseased hearts. Therefore, having the expression data analyzed across cardiac maturation would add more significance to this finding.**

To answer this question, we checked the expression of the minor spliceosome components throughout development in the following RNA-seq dataset: https://apps.kaessmannlab.org/evodevoapp/ (Cardoso-Moreira et al., 2019), which includes mRNA expression data of rat hearts at different stages from embryonic day E11 to postnatal day P112 (3.5 months). We have plotted the results in the figure below.

© 2021. Published by The Company of Biologists under the terms of the Creative Commons Attribution License (https://creativecommons.org/licenses/by/4.0/).

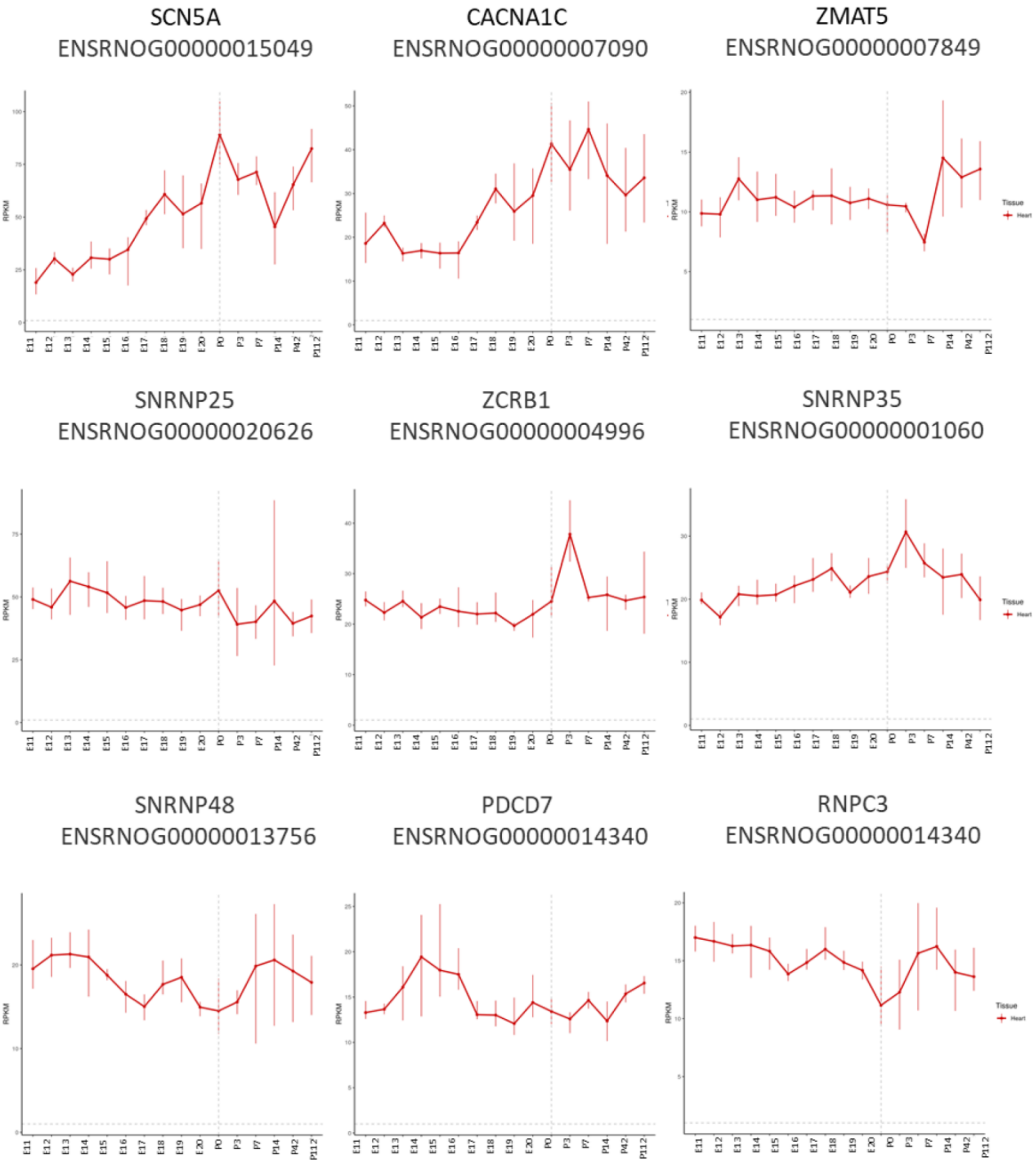

Gene expression of the minor-intron containing genes Scn5a and Cacna1c, and the core protein components of the minor spliceosome in the rat heart at different stages during development.

This analysis revealed that the mRNA expression of *Scn5a* and *Cacna1c* is increased during embryonic development, but when comparing the expression levels of p0 with p112 not much difference can be observed. Expression of minor spliceosome components throughout embryo development and adulthood was rather constant. Unfortunately, snRNAs were not available in this dataset. To our opinion, this analysis did not yield relevant findings and therefore we refrain from including this data to the manuscript.

6) It is interesting to note that PTEN is also a MIG and subjected to minor-intron retention upon minor-intron specific spliceosome inactivation. Since PTEN is a key molecule in PI3K/AKT signaling during cardiac hypertrophy, it would be very interesting to evaluate the impact on cardiomyocyte size and growth under hypertrophic stimulations. Authors measured confluency, morphology and apoptotic activity under normal growth condition. However, cell surface area can be measured under basal (serum starved) vs. post-hypertrophic stimulation (using PE or serum) to evaluate PTEN mediated hypertrophic regulation.

© 2021. Published by The Company of Biologists under the terms of the Creative Commons Attribution License (https://creativecommons.org/licenses/by/4.0/).

Thank you for this suggestion. We performed additional experiments to compare the hypertrophic response in NRVM transfected with either a non-targeting control or anti-*U6atac* gapmeR. After transfection and a 24h step of serum starvation, we incubated the NRVM for 48h in serum-free or in 2% Fetal Bovine Serum (FBS) medium. We quantified cell surfaces after staining the cell membrane with wheat-germen-aglutinin-488 (WGA), as a measure of hypertrophy. In short, without serum (0% FBS) we did not see any difference in cell size after knocking down *U6atac*. Serum stimulation with 2% FBS induced hypertrophy in both gapmeR groups, however the hypertrophic response was blunted in the anti-*U6atac* treated group. These results indicate that *U6atac* knockdown also affects cardiomyocyte hypertrophy, which may be mediated by *Pten* and other MIGs that participate in the hypertrophic response. These results were added as Fig 3C and to the text lines 183 to 191.

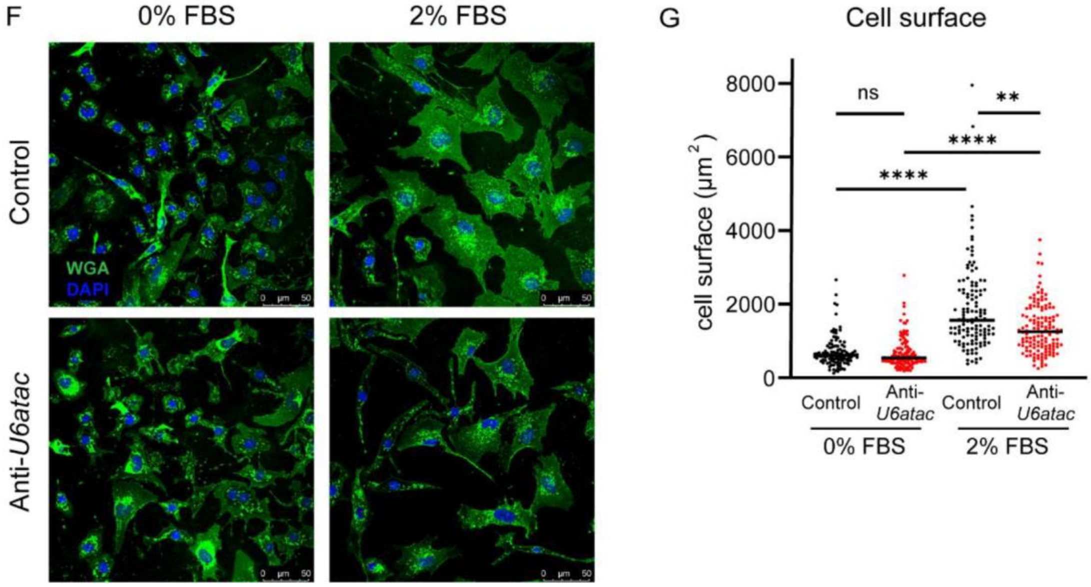

References

Cardoso-Moreira, M., Halbert, J., Valloton, D., Velten, B., Chen, C., Shao, Y., Liechti, A., Ascenção, K., Rummel, C., Ovchinnikova, S., Mazin, P.V., Xenarios, I., Harshman, K., Mort, M., Cooper, D.N., Sandi, C., Soares, M.J., Ferreira, P.G., Afonso, S., Carneiro, M., Turner, J.M.A., VandeBerg, J.L., Fallahshahroudi, A., Jensen, P., Behr, R., Lisgo, S., Lindsay, S., Khaitovich, P., Huber, W., Baker, J., Anders, S., Zhang, Y.E., Kaessmann, H., 2019. Gene expression across mammalian organ development. Nature 571, 505–509. https://doi.org/10.1038/s41586-019-1338-5

Diebold, R.J., Koch, W.J., Ellinor, P.T., Wang, J.J., Muthuchamy, M., Wieczorek, D.F., Schwartz, A., 1992. Mutually exclusive exon splicing of the cardiac calcium channel alpha 1 subunit gene generates developmentally regulated isoforms in the rat heart. Proc. Natl. Acad. Sci. 89, 1497–1501. https://doi.org/10.1073/pnas.89.4.1497

Hu, Z., Wang, J.-W., Yu, D., Soon, J.L., de Kleijn, D.P.V., Foo, R., Liao, P., Colecraft, H.M., Soong, T.W., 2016. Aberrant Splicing Promotes Proteasomal Degradation of L-type CaV1.2 Calcium Channels by Competitive Binding for CaVβ Subunits in Cardiac Hypertrophy. Sci. Rep. 6, 35247. https://doi.org/10.1038/srep35247

Kong, S.W., Hu, Y.W., Ho, J.W.K., Ikeda, S., Polster, S., John, R., Hall, J.L., Bisping, E., Pieske, B., dos Remedios, C.G., Pu, W.T., 2010. Heart Failure–Associated Changes in RNA Splicing of Sarcomere Genes. Circ. Cardiovasc. Genet. 3, 138–146. https://doi.org/10.1161/CIRCGENETICS.109.904698

Liao, P., Yu, D., Hu, Z., Liang, M.C., Wang, J.J., Yu, C.Y., Ng, G., Yong, T.F., Soon, J.L., Chua, Y.L., Soong, T.W., 2015. Alternative Splicing Generates a Novel Truncated Cav1.2 Channel in Neonatal Rat Heart *. J. Biol. Chem. 290, 9262–9272. https://doi.org/10.1074/jbc.M114.594911

Onkal, R., Mattis, J.H., Fraser, S.P., Diss, J.K.J., Shao, D., Okuse, K., Djamgoz, M.B.A., 2008. Alternative splicing of Nav1.5: An electrophysiological comparison of 'neonatal' and

© 2021. Published by The Company of Biologists under the terms of the Creative Commons Attribution License (https://creativecommons.org/licenses/by/4.0/).

‘adult’ isoforms and critical involvement of a lysine residue. J. Cell. Physiol. 216, 716–726. https://doi.org/10.1002/jcp.21451
Younis, I., Dittmar, K., Wang, W., Foley, S.W., Berg, M.G., Hu, K.Y., Wei, Z., Wan, L., Dreyfuss, G., 2013. Minor introns are embedded molecular switches regulated by highly unstable U6atac snRNA. eLife 2013. https://doi.org/10.7554/eLife.00780

Second decision letter

MS ID#: JOCES/2021/259191

MS TITLE: Inhibition of minor intron splicing reduces Na+ and Ca2+ channel expression and function in cardiomyocytes

AUTHORS: Pablo Montañés-Agudo, Simona Casini, Simona Aufiero, Auriane C. Ernault, Ingeborg van der Made, Yigal M. Pinto, Carol Ann Remme, and Esther E. Creemers
ARTICLE TYPE: Research Article

I am happy to tell you that your manuscript has been accepted for publication in Journal of Cell Science, pending standard ethics checks.

Reviewer 1

Advance summary and potential significance to field

This manuscript contributes to splicing field with minor intron splicing and its importance.

Comments for the author

The authors addressed my concerns and comments to a satisfactory level. I do not have any further comments on the manuscript.

Reviewer 2

Advance summary and potential significance to field

The authors have responded well to reviewer input

Comments for the author

The authors have responded well to reviewer input and this reviewer is satisfied

Reviewer 3

Advance summary and potential significance to field

The revision is comprehensive and responsive to the reviewers' comments. The report is improved in rigor and significance accordingly. Thank you for the efforts.

Comments for the author

This is an interesting and potentially relevant report on the molecular features as well as functional outcome of minor intron regulation in cardiomyocytes. The revision is quite responsive and improved with several additional data and analyses.

© 2021. Published by The Company of Biologists under the terms of the Creative Commons Attribution License (https://creativecommons.org/licenses/by/4.0/).
